# A New Cr^3+^ Electrochemical Sensor Based on ATNA/Nafion/Glassy Carbon Electrode

**DOI:** 10.3390/ma13122695

**Published:** 2020-06-12

**Authors:** Reda M. El-Shishtawy, Mohammed M. Rahman, Tahir Ali Sheikh, Muhammad Nadeem Arshad, Fatimah A. M. Al-Zahrani, Abdullah M. Asiri

**Affiliations:** 1 Chemistry Department, Faculty of Science, King Abdulaziz University, Jeddah 21589, Saudi Arabia; mnachemist@hotmail.com (M.N.A.); aasiri2@kau.edu.sa (A.M.A.); 2 Irrigation Research Institute, Irrigation Department, Government of the Punjab, Old Anarkali, Lahore-54000, Pakistan; tahirgcu786@gmail.com; 3 Chemistry Department, Faculty of Science, King Khalid University, P.O. Box 9004, Abha 61413, Saudi Arabia; falzhrani@kku.edu.sa

**Keywords:** 1,1′-(-((Disulfanediylbis(2,1-phenylene))bis(azaneylylidene))bis(methaneylylidene))bis(naphthalene-2-ol), homobifunctional tridentate disulfide Schiff base, Cr^3+^ ions, electrochemical method, glassy carbon electrode, environmental remediation

## Abstract

A new electrochemical sensor of metal cation in an aqueous solution based on homobifunctional tridentate disulfide Schiff base and named 1,1′-(-((disulfanediylbis(2,1-phenylene))bis(azaneylylidene))bis(methaneylylidene))bis(naphthalene-2-ol) (ATNA) was easily obtained quantitatively from the condensation reaction of 2-hydroxy-1-naphthaldehyde and *2*-aminothiophenol, and then fully characterized by spectroscopic techniques for structure elucidation. The molecular structure of ATNA was also confirmed by a single-crystal X-ray diffraction study to reveal a new conformation in which the molecule was stabilized by the O–H…N type intramolecular hydrogen bonding interactions in both moieties. The ATNA was used as a selective electrochemical sensor for the detection of chromium ion (Cr^3+^). A thin film of ATNA was coated on to the flat surface of glassy carbon electrode (GCE) followed by 5 % ethanolic Nafion in order to make the modified GCE (ATNA/Nafion/GCE) as an efficient and sensitive electrochemical sensor. It was found to be very effective and selective against Cr^3+^ cations in the company of other intrusive heavy metal cations such as Al^3+^, Ce^3+^, Co^2+^, Cu^2+^, Ga^3+^, Hg^2+^, Mn^2+^, Pb^2+^, and Y^3+^. The detection limit at 3 S/N was found to be 0.013 nM for Cr^3+^ ions within the linear dynamic range (LDR) (0.1 nM–10.0 mM) of Cr^3+^ ions with r^2^ = 0.9579. Moreover; this work instigates a new methodology for developing the sensitive as well as selective electrochemical toxic cationic sensors in the field of environmental and health care.

## 1. Introduction

The chemical factories and mining facilities throughout the world discharge overwhelming metal particles and severely affect the environment [1,2,3,4]. Hence, accurate quantitative and qualitative detection of such heavy metal ions in an aqueous solution is of prime importance for the protection of the environment. In this regard, the Schiff base structure represents an extraordinary class of ligands because of their facile synthesis from diverse reagents, and the formation of a wide range of complexes with potential applications in different fields such as dye affinity chromatography [5], liquid crystals [6,7], nanocomposite-based polyazommethine [8], medicine [9,10], corrosion inhibitors [11], catalysis [12] and electrochemical sensors [13,14,15]. Schiff base-derived from 2-aminobenzothiophenol, on the other hand, may have an interesting geometry owing to its propensity for the in situ formed disulfide bond [16]. Accordingly, the present work was made to explore the possibility of synthesizing and using of homobifunctional tridentate disulfide Schiff base derived from 2-hydroxy-1-naphthaldehyde and *2*-aminothiopheno as a selective cation sensor by an electrochemical approach. 

Chromium, among the most well known components, that exists in the earth crust as Cr^3+^ or Cr^6+^ cations in combination with other anions as ore e.g., chromite. Cr^3+^ is a vital component in nutrition, while Cr^6+^ is profoundly harmful. Cr^3+^ plays a vital role during metabolism via enzyme activation [17,18,19]. On the other hand, Cr^3+^ deficiency in the human body may cause health disorders, including diabetes and cardiovascular diseases. However, a high level of chromium ion in the human body is very harmful as it affects the enzymatic activities and the cellular structures [20]. The maximum permissible level of total chromium, as set by the Environmental Protection Agency (EPA) is 0.1 mg/mL [18,21,22]. 

Several analytical techniques, for example, atomic absorption spectrometry (AAS) [23,24,25,26,27], high-performance liquid chromatography (HPLC) coupled with inductive coupled plasma–mass spectrometry (HPLC–ICP–MS), high-performance liquid chromatography coupled with diode array detector (HPLC–DAD), reverse phase ion-pair high-performance liquid chromatography [28,29,30,31,32,33], ion chromatography inductively coupled plasma–mass spectrometry (IC–ICP–MS) [34,35], mass spectrometry [31], colorimetric and fluorescence spectroscopy [36,37,38,39], capillary electrophoresis coupled with UV spectrophotometer [40,41], and chemiluminescence [42] have been reported for the detection of chromium ionic species in addition to some electrochemical approaches such as differential pulse voltammetry [43], adsorptive stripping voltammetry [44], cyclic voltammetry and amperometry [45,46,47,48]. Some of the methods that have been stipulated above are very expensive and complicated for the detection of chromium. If some are fortunately cost effective, then they are not effectual with respect to the detection limit, and some exist with other old classical electrochemical methods for its detection. 

Therefore, it is of great interest to develop a new, trustworthy, efficient, selective and cost-effective (cheap) techniques for the detection of chromium ion (Cr^3+^), qualitatively, and quantitatively. In this study, an electrochemical approach, current-potential (I-V) technique, based on newly modified glassy carbon electrode, 1,1′-(-((disulfanediylbis(2,1-phenylene))bis(azaneylylidene))bis(methaneylylidene))bis(naphthalene-2-ol) (ATNA)/Nafion/GCE, as an agile and selective electrochemical sensor, was applied for the first time for the practical determination of Cr^3+^ ions in an aqueous media (phosphate buffer saline (PBS)). The modified GCE (ATNA/Nafion/GCE) was fabricated by coating the newly synthesized, homobifunctional tridentate ATNA Schiff base, which acts as a chelating agent onto its flat surface followed by 5% ethanolic Nafion as conducting the polymer binder. It furnishes the very sensitive transduction at the interface of liquid/surface electrode in phosphate buffer saline (PBS) (0.1 M at 7.0 pH). To the best of our knowledge, this is the first cationic electrochemical sensing application based on the current-potential technique by using a newly modified GCE as a working electrode for sensing the Cr^3+^ ions within a very short response time of 10 to 20 s, qualitatively and quantitatively, in an aqueous neutral solution.

## 2. Experimental

### 2.1. Materials and Methods

All solvents and reagents were purchased from Sigma-Aldrich Company and used as received. Then, 1 H and 13C NMR spectra were recorded in CDCl_3_ solutions on a Bruker Avance 850 MHz spectrometer. The attenuated total reflectance–Fourier transform infrared (ATR–FTIR) spectrum was performed on a PerkinElmer spectrum 100 FT-IR spectrometer. The melting point was determined in an open capillary tube in a Stuart Scientific melting point apparatus (SMP3) and was uncorrected. De-ionized water was used for the making the stock as well as the chemical solutions of different concentration in whole study.

The current-potential (*I-V*) technique was measured using the Keithley 6517A, Electrometer (USA), as a constant voltage source. The detection of chromium cation (Cr^3+^) in the phosphate buffer medium at applied potential ranging from 0 to + 1.5 V was made using a newly designed ATNA/Nafion/GCE and Pt-wire as the working electrode and counter electrode, respectively, by measuring the I-V responses in two electrode systems (lab-made electrochemical cell). Moreover, this novel (*I-V*) method follows the Ohms law that measures the current against the potential applied.

### 2.2. Synthesis of 1,1′-(-((disulfanediylbis(2,1-phenylene))bis(azaneylylidene))bis(methaneylylidene))bis(naphthalen-2-ol) (ATNA)

A solution of 2-aminothiophenol (0.88 g, 7.0 mmol) and 2-hydroxy-1-naphthaldehyde (1.21 g, 7.0 mmol) in 50.0 ml absolute ethanol was refluxed with stirring overnight, and then left to cool. The brownish-orange precipitate was filtered and then washed with ethanol to give 1.8 g, 92.5% yield. The product was re-crystallized slowly from toluene to obtain the single crystal. Mp: 221–220 °C. ^1^H NMR (CDCl_3_, 850 MHz,δ = ppm): 7.20 (6H, m, Ar-H), 7.27 (2H, d, J = 8.5 Hz, ArH), 7.40 (2H, t, J = 7.65–8.5 Hz, Ar-H), 7.56 (2H, t, J =7.65–8.5 Hz, Ar-H), 7.73 (2H, d, J = 7.65 Hz, Ar-H), 7.80 (2H, d, J = 7.65 Hz, Ar-H), 7.89 (2H, d,J = 8.5 Hz, Ar-H), 8.19 (2H, d, J = 8.5 Hz, Ar-H), 9.45 (2H, s, 2N = CH), 14.95 (2H, s, 2OH), ^13^C NMR (CDCl_3_, 213 MHz, δ = ppm):109.56, 118.08, 119.31, 120.60, 123.69, 127.30, 127.76, 128.06,128.36, 129.07, 129.39, 130.98, 132.99, 135.98, 146.45, 157.55, 157.61; FTIR (ATR, cm^−1^): 3061, 3001, 2972, 1622, 1602, 1586, 1551, 1461, 1217, 819, 744, 449.

### 2.3. X-ray Crystallography of ATNA

The sample compound was crystalized for single crystal diffraction studies. The crystals were taken out from the crystallization flask and screened out under the microscope. The suitable crystal was selected and pasted over the glass tip using commercial glue. The glass tip was immersed into the wax supported by a hollow copper rod and a magnetic base. This holder was mounted on an Agilent SuperNova (Dual source) Agilent Technologies Diffractometer, equipped with graphite-monochromatic Cu/Mo Kα radiation for data collection. The data collection was accomplished using CrysAlisPro software [49] at 296 K under the Cu Kα radiation. The structure solution was performed using SHELXS–97 [50] and refined by full-matrix least-squares methods on *F*^2^ using SHELXL–97 [50], in-built with WinGX [51]. All non-hydrogen atoms were refined anisotropically by full-matrix least squares methods [50]. Figures were drawn using PLATON [52] and *ORTEP*-3 [53].

All the aromatic hydrogen atoms were positioned geometrically and treated as riding atoms with C–H = 0.93 Å and Uiso(H) = 1.2 Ueq(C) carbon atoms. The O–H = 0.82 Å and O–H = 0.93(6) Å, hydrogen atom was also positioned geometrically and treated as a riding atom with Uiso (H) = 1.5 Ueq(O), respectively. The crystallographic information file (CIF) of compound I was submitted to the Cambridge Crystallographic Data Centre (CCDC). The CCDC number (1953254) obtained against it is mentioned in Table 1. These data can be obtained free of charge at www.ccdc.cam.ac.uk/conts/retrieving.html or from the Cambridge Crystallographic Data Centre, 12 Union Road, Cambridge CB2 1EZ, UK.

### 2.4. Fabrication of ATNA/Nafion/GCE as a Selective Cr^3+^ Electrochemical Sensor

**Scheme 1:**
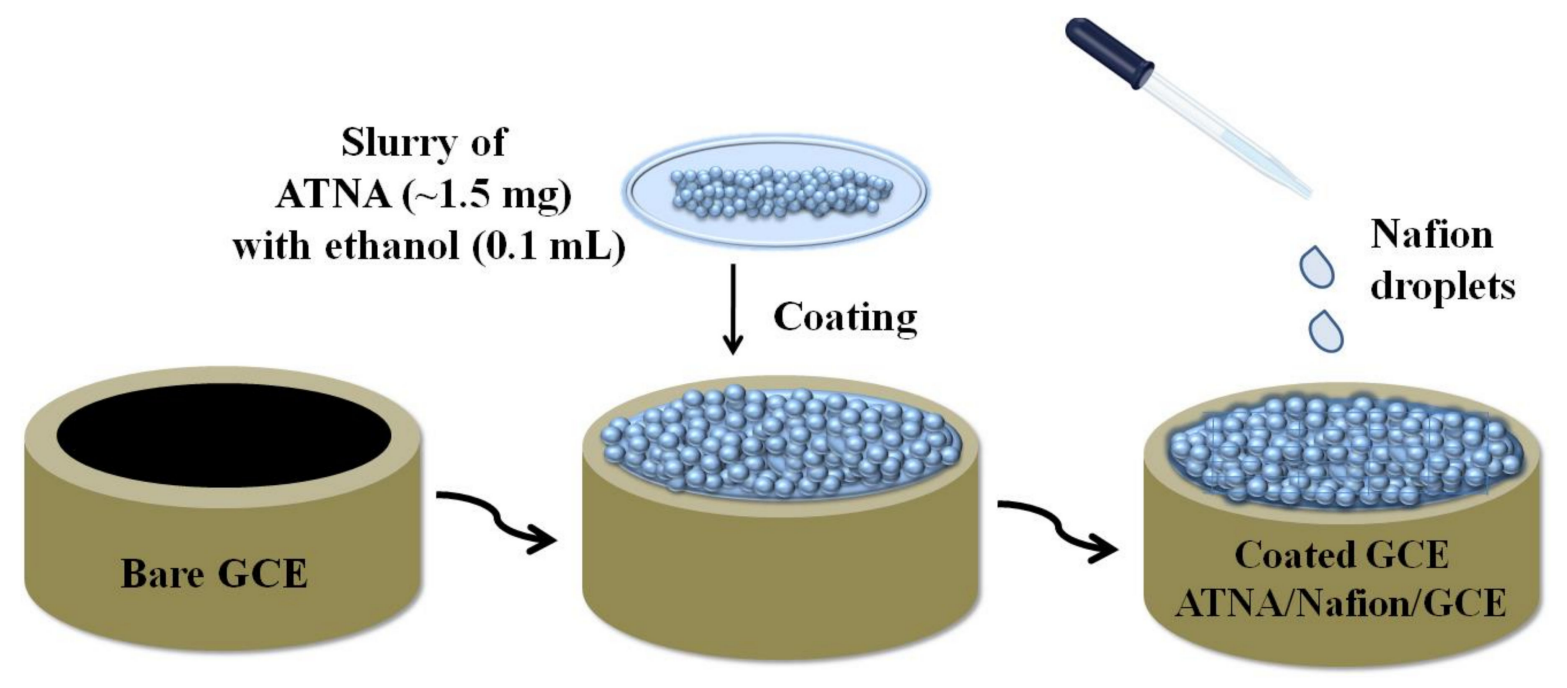
Fabrication of glassy carbon electrode GCE modified by, 1′-(-((disulfanediylbis(2,1-phenylene))bis(azaneylylidene))bis(methaneylylidene))bis(naphthalene-2-ol) (ATNA) with the conducting binder, 5% Nafion.

For the fabrication of ATNA/Nafion/GCE as a selective Cr^3+^ electrochemical sensor, a simple and reliable approach was practiced. The geometrical dimension of the GCEs that were used for the fabrication of GCE as a selective Cr^3+^ electrochemical sensor, was 12 cm in length with a copper rod and 0.0316 cm^2^ in surface area. Before the fabrication of the GCE, it was washed by using a very simple protocol. First, the GCE was dipped in acetone for 10 min. After that, it was washed thoroughly with deionized water followed by ethanol-soaked cotton buds and dried in an oven for 15 min at 80 ºC. After proper washing, a slurry of approximately 1.5 mg of synthesized ATNA was made with ethanol, and then applied onto the flat surface of GCE with one to two drops of 5% ethanolic Nafion as an adhesive conducting binder. Then, the freshly fabricated electrode was dried at 35 ºC for 45 min in an oven in order to get the ATNA/Nafion/GCE as a selective and sensitive Cr^3+^ electrochemical sensor, Scheme 1. This newly designed ATNA/Nafion/GCE as a selective Cr^3+^ sensor was therefore used as a working electrode to measure the I-V (current-potential) response in a phosphate buffer saline (PBS) medium of pH = 7, in the presence or absence of Cr^3+^ ions, in a lab-made electrochemical cell (beaker) in which Pt-wire was used as a counter electrode. The (PBS) medium of pH = 7 was made by mixing the equimolar solution of disodium phosphate (0.2 M, 39.0 mL) with monosodium phosphate (0.2 M, 61.0 mL) in a 100 mL measuring flask. In the whole study, Cr^3+^ was used as a target analyte in the presence of other interference cations. The stock solution of Cr^3+^ was used to get the different concentration range of Cr^3+^ (full concentration range: 0.01 nM ~ 0.1 M) in deionized water by dilution method. Similarly, the amount (10.0 mL) of (0.2 M) PBS of pH = 7 was kept constant in every trial or throughout the whole study in a lab-made electrochemical cell in order to measure the I-V response, in the presence as well as in the absence of the target analyte. Correspondingly, different analytical parameters such as sensitivity, full concentration range as well as linear dynamic range (LDR), regression coefficient r^2^, the limit of detection at S/N 3 (LOD) and limit of quantification (LOQ), for Cr^3+^ were also measured from the slope of the calibration plot (current versus concentration) by using the newly designed ATNA/Nafion/GCE as a selective Cr^3+^ sensor.

## 3. Results and Discussion

### 3.1. Synthesis

**Scheme 2:**
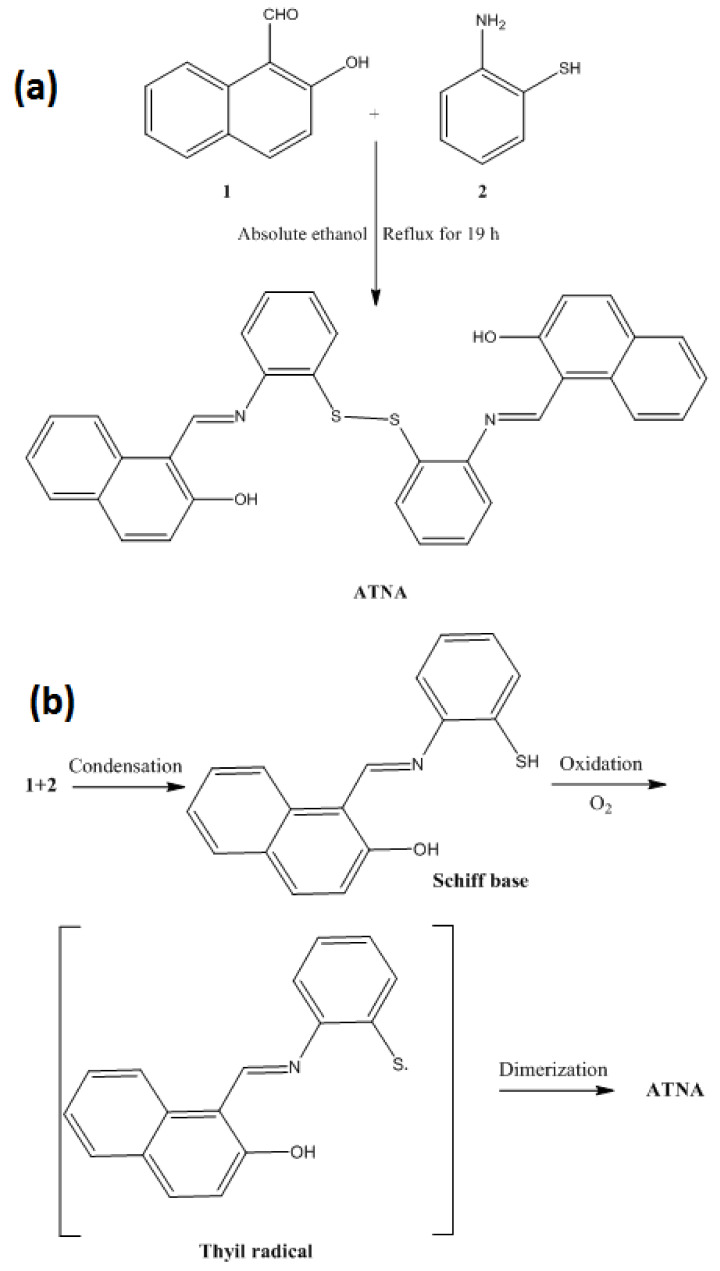
(**a**) Synthesis of ATNA. (**b**) Suggested mechanism for the formation of ATNA.

It was envisioned that having disulfide-containing homobifunctional Schiff base as a tridentate ligand would make a stable film on a glassy carbon electrode for the electrochemical detection of toxic metal ions from aqueous solution. Little information on ATNA with a different single-crystal structure from the present one has been reported [16]. The synthesis of ATNA was easily obtained in one step in excellent yield by refluxing the 1:1 molar ratio of 2-aminothiphenol and 2-hydroxynaphthaldehyde in absolute ethanol for 19 h (Scheme 2a). The single crystal of the product was obtained by recrystallization from toluene. It is suggested that the formation of the ATNA ligand proceeds via condensation to get the corresponding Schiff base, which further undergoes the in situ oxidation of thiol to the corresponding disulfide by virtue of the dissolved oxygen. The oxidation proceeds via a thyil radical in a one-electron transfer process that dimerizes to give the disulfide (Scheme 2b) [54].

**Scheme 3:**
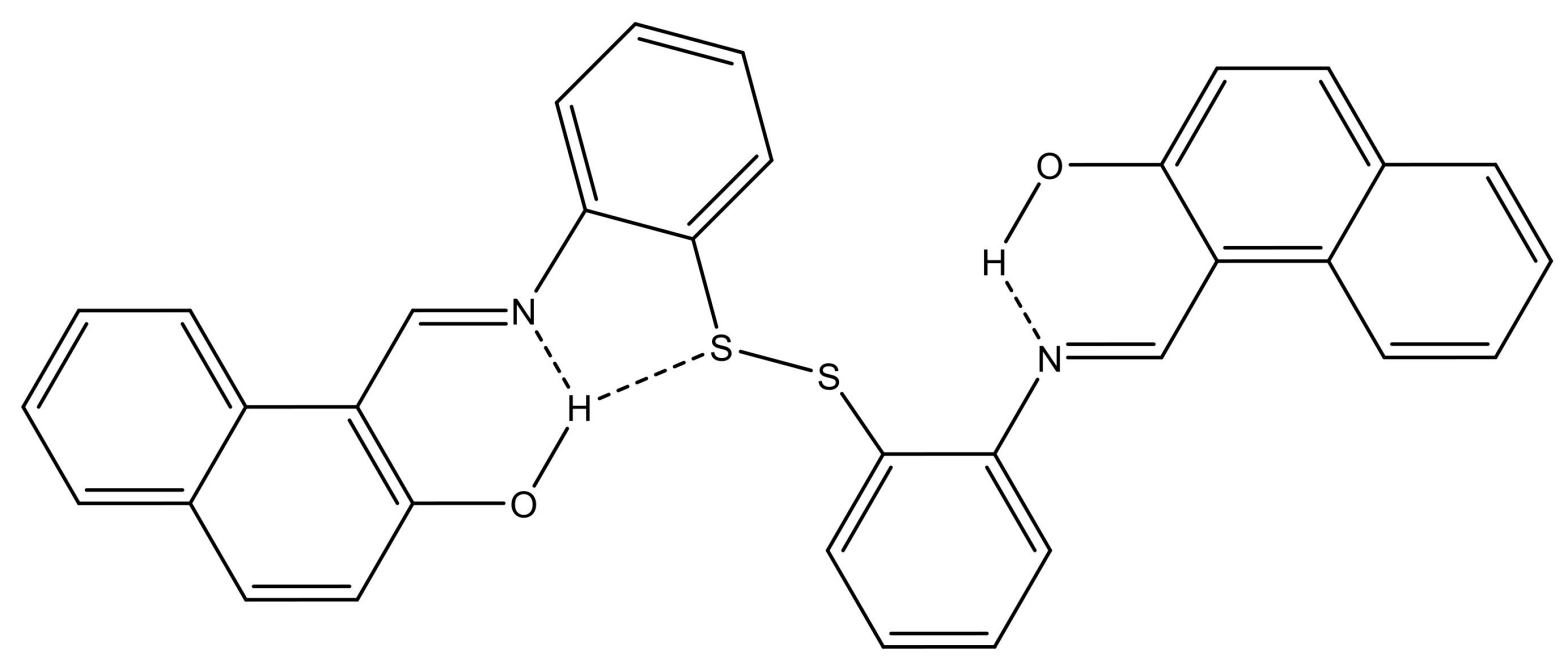
Intramolecular hydrogen bonds in ATNA.

**Figure 1:**
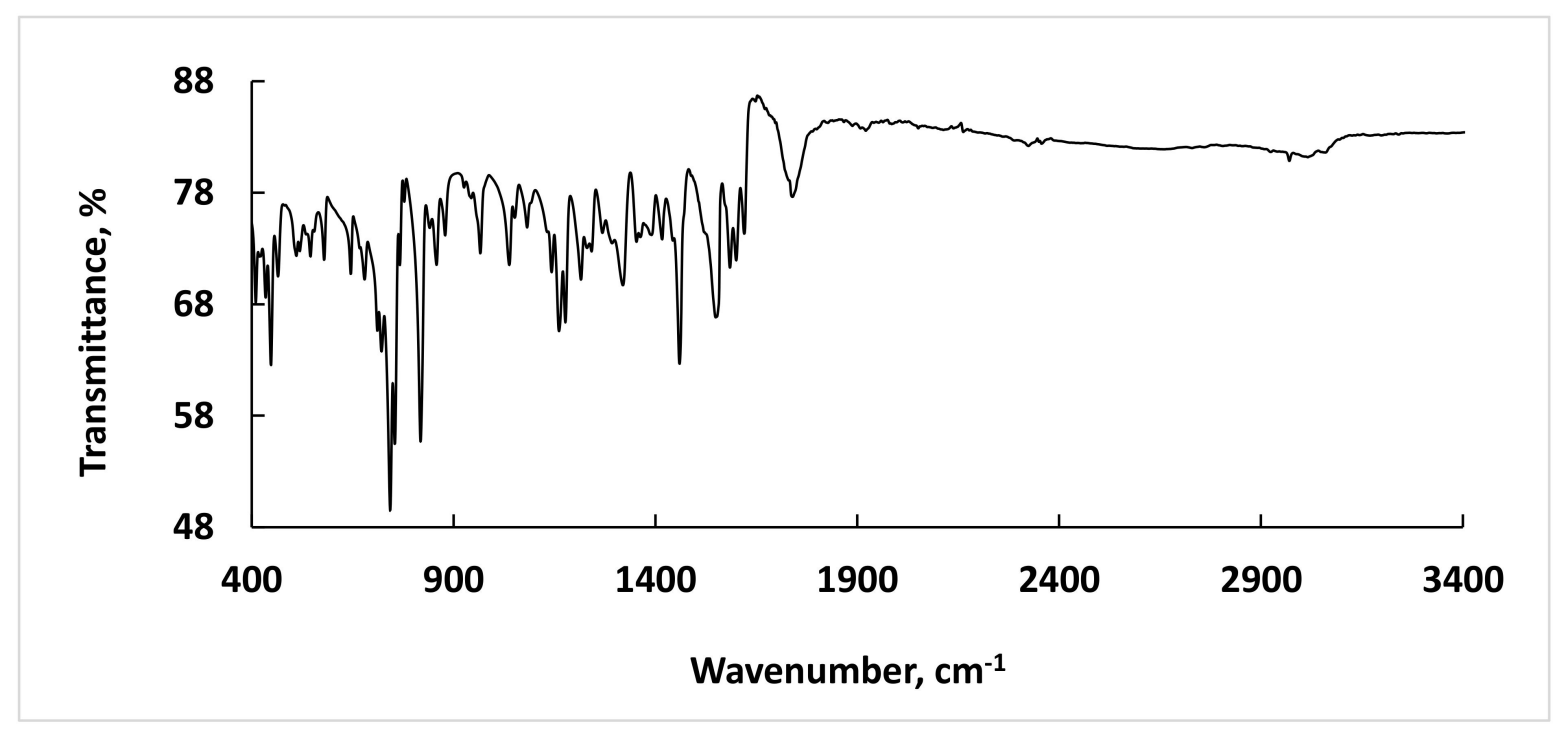
Attenuated total reflectance–Fourier transform infrared (ATR–FTIR) of the ATNA molecule.

NMR (proton and carbon, see Supplementary Files S1–S6, Figures S1 and S2), ATR–FTIR, and single-crystal measurements were made to confirm the chemical structure of ATNA. The ATR–FTIR spectrum of ATNA reveals the characteristic bands of aromatic Schiff base imine group (CH=N) at 1622 cm^−1^. It is noteworthy to observe, as shown in Figure 1, the absence of the vibration peak of the OH group, but instead the appearance of weak bands at 2972, 3001, 3061 cm^−1^ due to the stretching vibrations of C–H of imine and phenyl groups. The absence of OH vibration is attributed to the existence of the intramolecular hydrogen bond between OH and CH=N and S–S (Scheme 3), as it will be emphasized further below in ^1^H NMR and single-crystal data. This IR attribution is in accordance with a similar Schiff base reported earlier [14,15,55]. The stretching vibration frequency of aromatic disulfide (S–S) appears at 449 cm^−1^ as similarly reported [56]. The sharp band appearing at 744 cm^−1^ may correspond to disulfides (C–S stretch) and 1,2-disubstituted aromatic. The band at 819 cm^−1^ corresponds to the aromatic C–H bending vibration [56]. Moreover, the stretching vibrations due to C=N- appear at 1602 and 1586 cm^−1^. On the other hand, bands due to C=C aromatic and C-0 of phenolic groups appear at 1551, 1461, and 1217 cm^−1^, respectively.

The proton NMR data reveals the imine protons as a singlet at 9.45 ppm and OH groups as singlet downfield at 14.95 ppm because of the existence of intramolecular hydrogen bonds that increase the acidity of OH groups. Furthermore, the formation of such intramolecular hydrogen bonds near aromatic moieties may deshield the OH groups by magnetic anisotropic effect [14,15]. Other protons with their coupling constants are nicely correlated, and the total number of protons are presented per the chemical structure of ATNA. In addition, ^13^C NMR reveals all the carbons present in ATNA and the characteristic peak of imine carbon appears at 165.49 ppm.

### 3.2. Crystal Description

**Figure 2:**
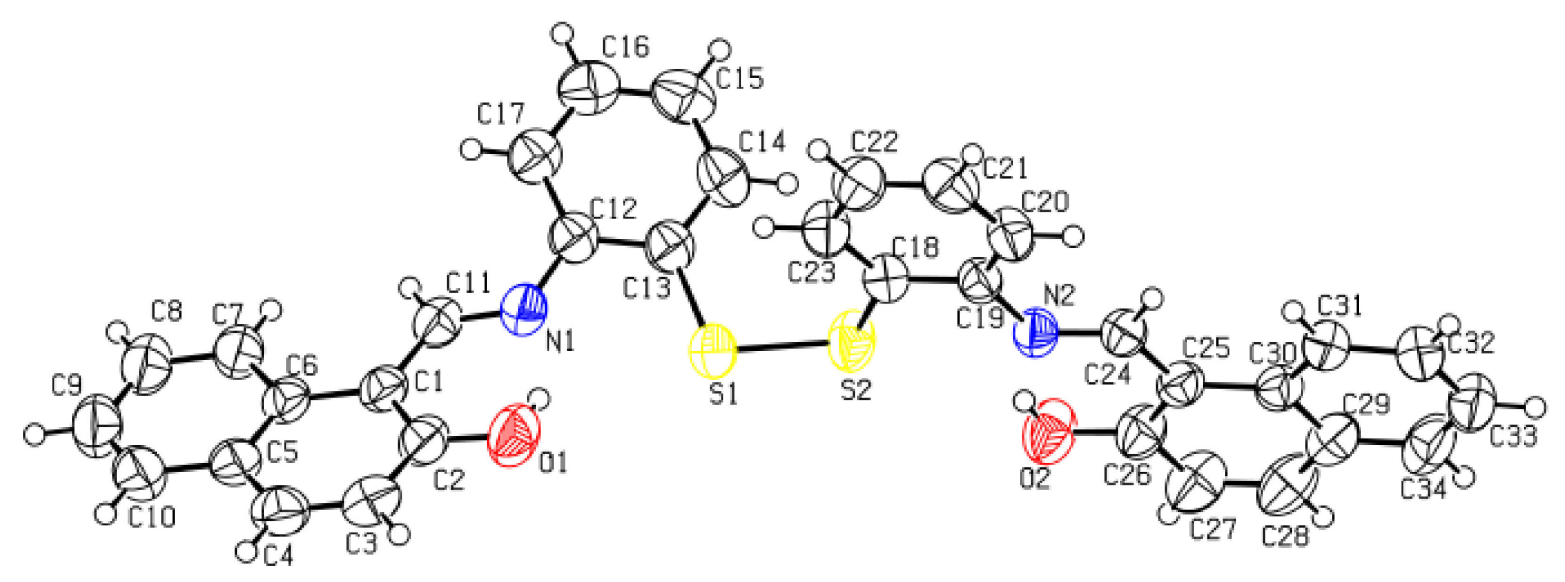
Plot for ATNA with 50% probability of thermal ellipsoids.

**Figure 3:**
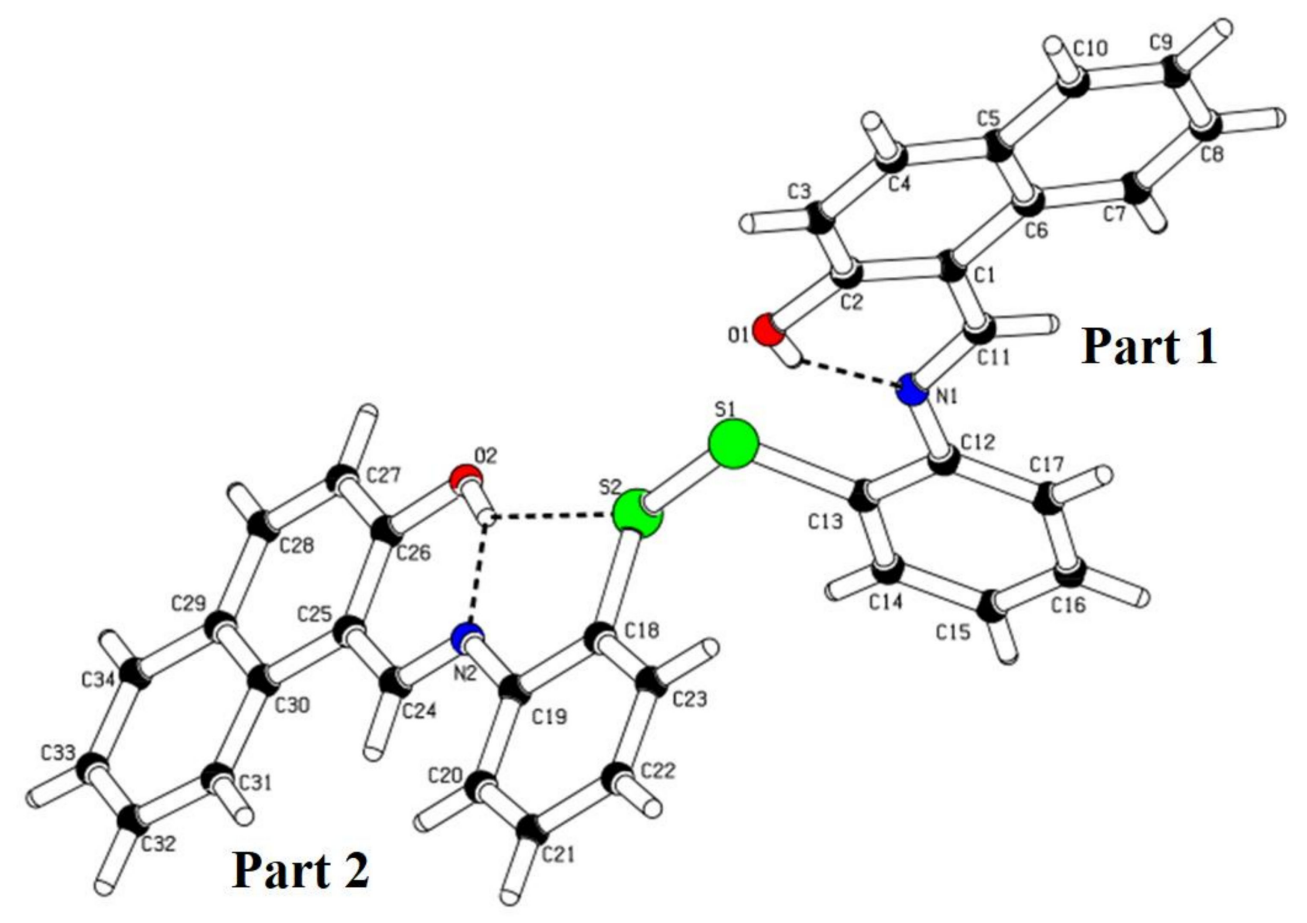
A labeled diagram showing the intramolecular interactions and the formation of different ring motifs.

The molecule 1,1′-(-((disulfanediylbis(2,1-phenylene))bis(azaneylylidene))bis (methaneylylidene))bis(naphthalen-2-ol) is basically a dimer of 1-[(2-Mercapto-phenylimino)-methyl]-naphthalen-2-ol which are connected through a sulphur atom. The single crystal diffractions were carried out to support our spectroscopic data, the confirmation of the synthesized product and to attract the readers for further any applications. The S–S bond distance is 2.0234 (10) Å, which is in accordance with already published related structures [57]. The Schiff base parts (A and B) of the molecule around the S–S bonds have a different geometry, due to which there is no center of symmetry or point symmetry in it, Figure 2. The crystallographic parameters are given in Table 1, while selected bond lengths and bond angles are provided in Tables S1 and S2. In part A (C1–C17), the benzene ring and naphthalene rings were twisted at a dihedral angle of 2.396(1)° while the in part B (C18–C34), these rings were twisted at a dihedral angle of 28.396 (1)°. The conformation around the imine bond (C=N) in both parts is trans, favoring the molecule to stay in the most stable form. The molecule was stabilized by the O–H…N type intramolecular hydrogen bonding interactions (Table 2). These interactions produced six-membered ring motifs [*S*(6)] with the root mean square deviation values of 0.0250 (2)Å and 0.0190 Å for the rings (C1/C2/C11/N1/H1O/O1) and (C24/C25/C26/N2/H2O/O2). The former ring motif is turned at dihedral angles of 79.72(7) with respect to the benzene ring (C12–C17) while the later ring is twisted by 4.071(15) with the aromatic ring (C18–C23) Figure 3. The O2–H2O…N2 and O2–H2O…S2 interactions produced two ring motifs (Figure 3 and Figure S3), (i) a nine-membered ring motif (C18/C19/N2/C24/C25/C26/O2/H2O/S2) which can be represented mathematically as *S*(9) [58], (ii) a five-membered ring motif where O2 acts as donor atom via H2O to N2 and S2 so this can be represented as *S*_1_^2^(5). From Figure S4, we can observe that planes of aromatic benzene and naphthalene are parallel to each other give rise to the π–π interactions among them to afford the extra stability to the crystal structure of the subjected compound. 

### 3.3. Application: Detection of Cr^3+^ Cation with ATNA Modified GCE 

The most inspiring application of the homobifunctional tridentate ATNA Schiff base is the selective electrochemical sensing of Chromium (III) cations in an aqueous system in the presence of other interfering toxic/heavy metal cations. An electrochemical current-potential (I-V) approach based on a newly modified GCE (ATNA/Nafion/GCE) was applied for the first time for the sensing of Cr^3+^ ions in a lab-made electrochemical cell (two-electrode system accommodating a working electrode and a counter electrode in an aqueous solution). Moreover, the fabrication of the newly modified GCE has already been discussed in the experimental section of this study. Therefore, the fabrication of ATNA/Nafion/GCE for the probe of Cr^3+^ ions in an aqueous solution by means of the (I-V) technique is the new approach. It is highly efficient as well as a selective electrochemical sensor against the Cr^3+^. Moreover, no other reports have been cited in the literature by using this newly designed ATNA/Nafion/GCE as a selective Cr^3+^ electrochemical sensor. A remarkable variation in the current response was observed against the potential applied (0 to +1.5 V) in response to the absorbed analyte, Cr^3+^ onto the surface of the modified GCE. During the whole study, the concentration of our target analyte, as well as other heavy metal cations, were taken as 25.0 µL of 0.1 µM through a micropipette. After the fertile change in the current response, analytical parameters such as LDR, LOD, and LOQ were calculated from the calibration curve so as to optimize the ATNA/Nafion/GCE sensor as a sensitive and selective electrochemical sensor for the detection of Cr^3+^, qualitatively and quantitatively. 

**Figure 4:**
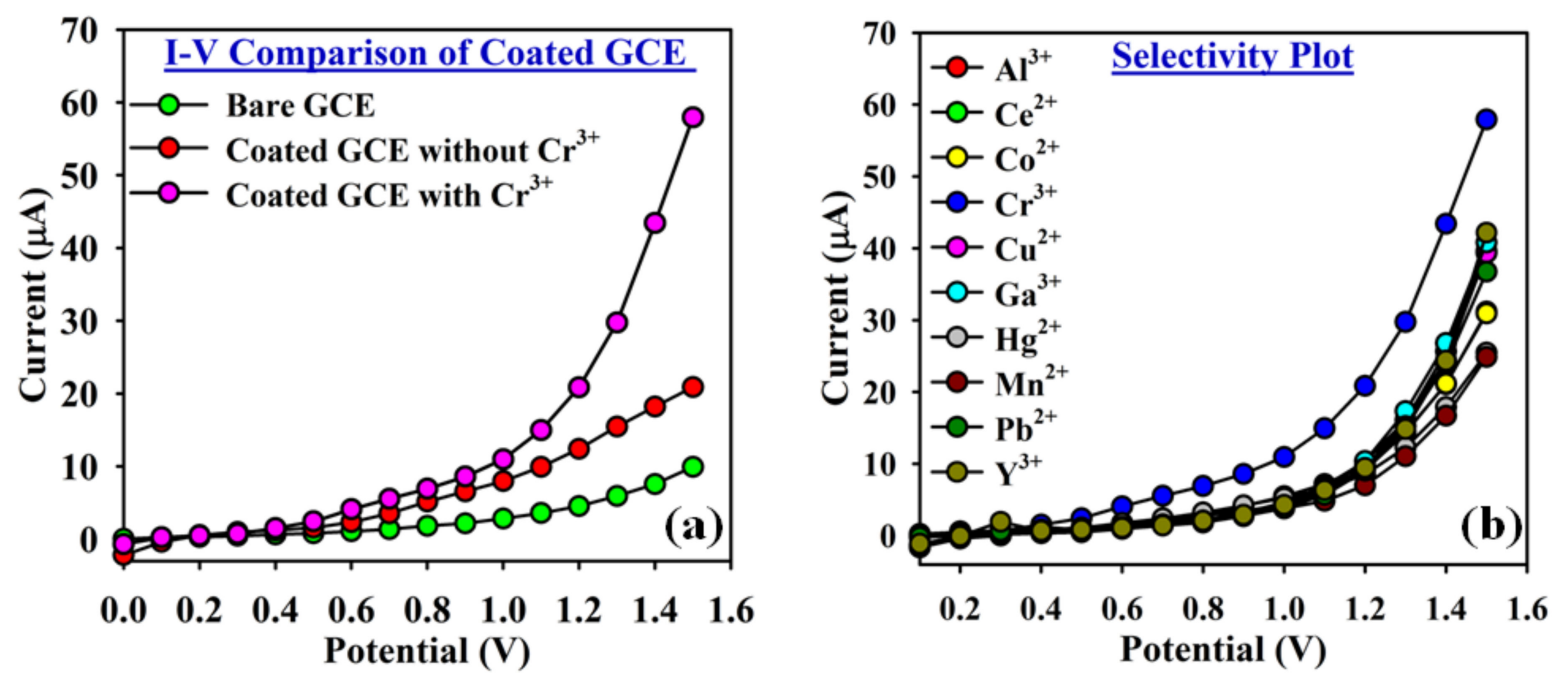
Selectivity study: (**a**) current potential (I-V) response of the bare and coated GCE (ATNA/Nafion/GCE) in the presence and absence of Cr^3+^ ions as our target analyte at 0.1 µM, 25.0 µL; (**b**) *I-V* response with various analytes (showing affinity with Cr^3+^), concentration of each analyte was taken at (0.1 µM, 25.0 µL).

At first, a change in the current response of the modified GCE (ATNA/Nafion/GCE) against the potential applied was recorded and evaluated with bare GCE. It was observed, that the ATNA/Nafion/GCE shows the high current response in contrast to a non-modified GCE due to the sensitive and excellent communication of the electron transduction in a PBS medium at the liquid/surface electrode interface, Figure 4a. After that, a selectivity study was conducted, and it was found to be very selective against the Cr^3+^ ions in the presence of other heavy metal cations such as Al^3+^, Ce^3+^, Co^2+^, Cu^2+^, Ga^3+^, Hg^2+^, Mn^2+^, Pb^2+^, and Y^3+^, Figure 4b. After the confirmation of selectivity of a newly modified GCE (ATNA/Nafion/GCE) against the Cr^3+^ ions, then a change in the current response of ATNA/Nafion/GCE against the potential applied was also checked in the presence and absence of a Cr^3+^ ion in order to check its affinity for Cr^3+^ ions. It was noticed that newly modified GCE gives a high response in the presence of Cr^3+^ ions as compared to its response in the absence of Cr^3+^ ions in PBS of pH = 7, Figure 4a. 

**Figure 5:**
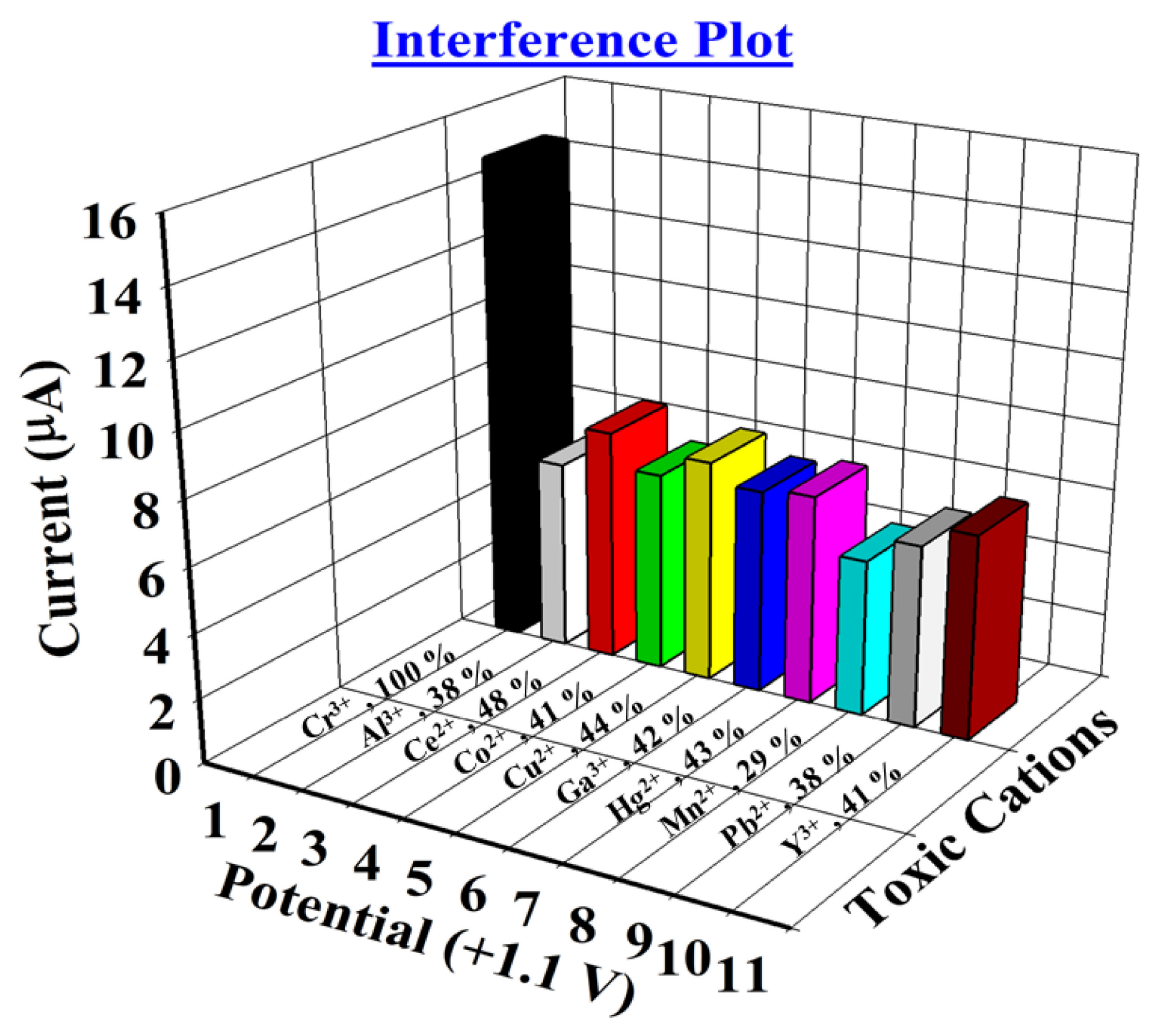
Interference study: comparison of the I-V responses of the interfering heavy metal cations at potential (+1.1 V). Analytes concentrations were taken as 25 µL of 0.1 µM, delay time one second.

After the selectivity, the interference study was conducted by using a statistical approach. The interference effect, in the presence of Cr^3+^ ions and of other heavy metal cations (Al^3+^, Ce^3+^, Co^2+^, Cu^2+^, Ga^3+^, Hg^2+^, Mn^2+^, Pb^2+^, and Y^3+^) on ATNA/Nafion/GCE was checked by measuring the I-V response at +1.1 V at STP and shown in Figure 5 and Table 3. The concentration of each analyte was taken at (0.1 µM, 25 µL) in PBS medium of pH =7. By this experiment, it was concluded that the modified GCE (ATNA/Nafion/GCE) is very selective for the Cr^3+^ ions, and it does not have any impressive impact towards the other above stipulated interfering heavy metal cations. Therefore, Figure 4b (selectivity) as well as Figure 5 (interference study) show that our synthesized compound has more affinity with Cr^3+^. Moreover, our synthesized ATNA also shows very little affinity with other heavy metal ions but these metal ions showed the similar current response against the potential applied up to +1.0 V but still cannot be distinguished with each other.

**Figure 6:**
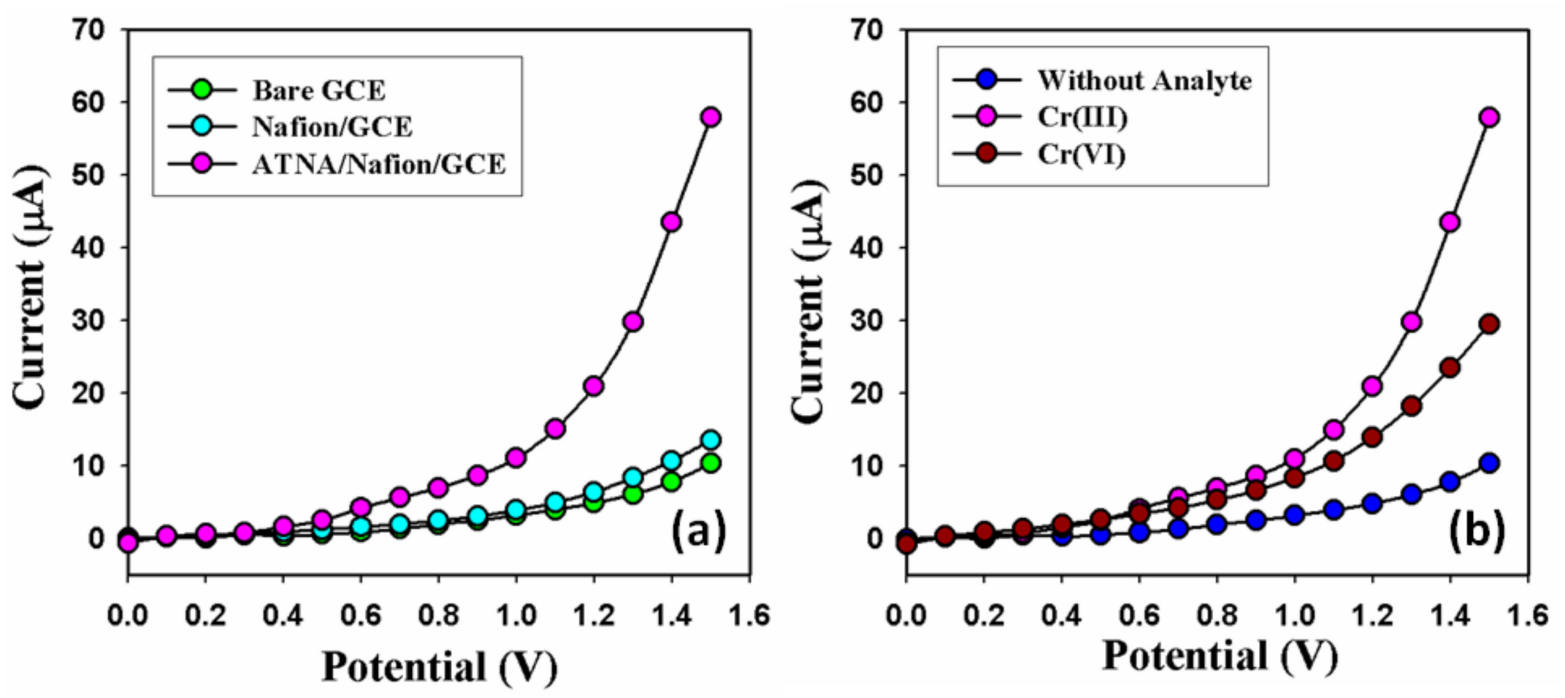
Control experiment: (**a**) the electrochemical responses of the bare GCE and the Nafion-coated GCE; and (**b**) the electrochemical responses of ATNA/Nafion/GCE in the presence of Cr^3+^ and Cr(VI).

A control experiment was also performed to check the effect of Nafion coating onto GCE. For this purpose, it was compared the electrochemical investigation with only GCE and Nafion/GCE were presented in Figure 6a. Here, it was observed that in presence of Nafion coating, the sensor signal did not have any significant changes compared to only the bare GCE in identical conditions. The Nafion/GCE electrode exhibited an almost similar current like bare GCE, and no significant changes in the current were observed. In this study, Nafion is used as a conducting coating binders to stick the ATNA materials onto the GCE for electrochemical analysis. Additionally, we performed a control experiment with both tri- and hexa-valent chromium as well as without analyte by ATNA/Nafion/GCE sensor probe and included the results in Figure 6b. Here, Cr(VI) shows less electrochemical response compared to the Cr^3+^ in identical conditions. Therefore, in this investigation, it was selected as the only Cr^3+^ sensor probe development based on ATNA/Nafion/GCE by using the selectivity study in the electrochemical investigation.

**Scheme 4:**
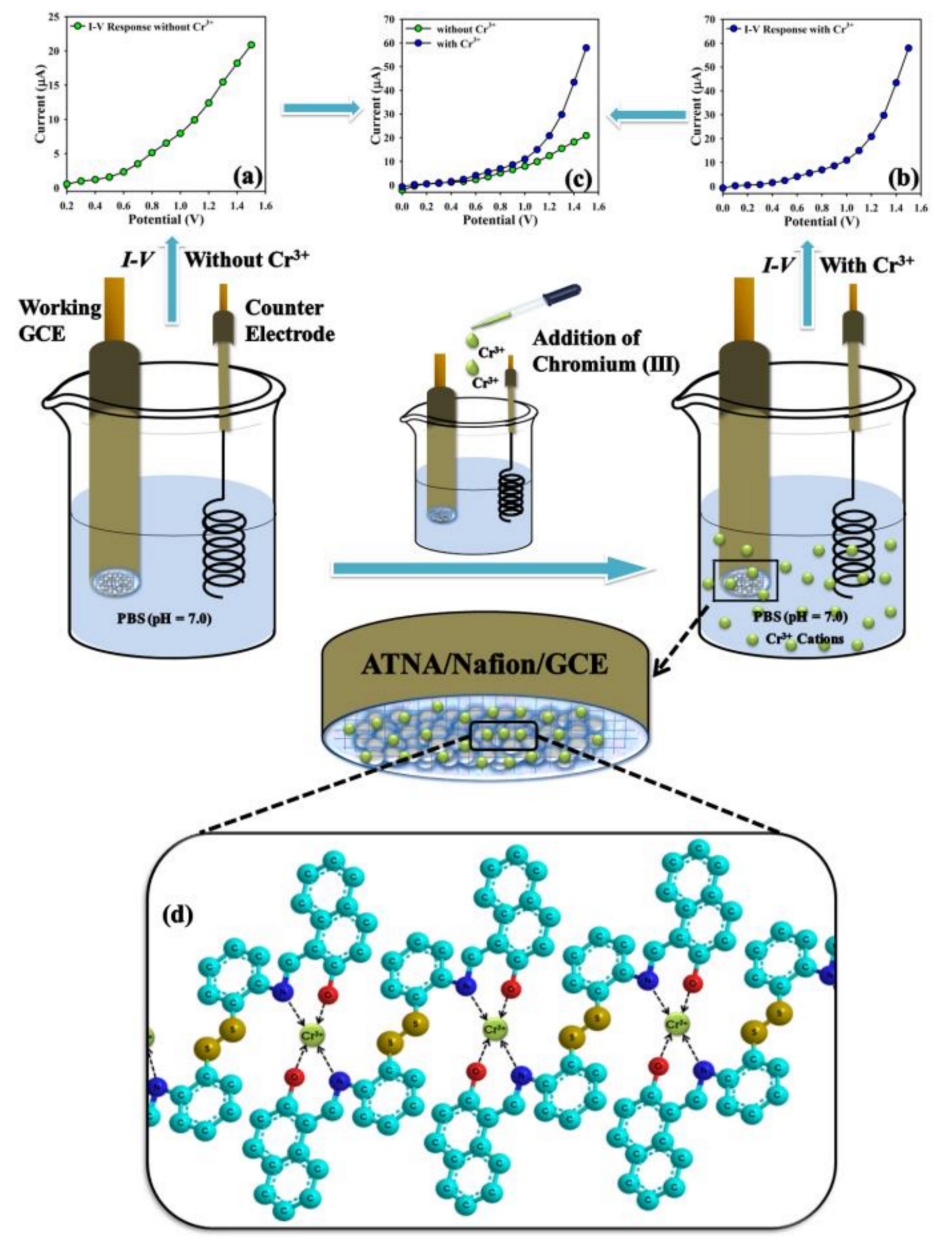
Proposed mechanism of the probable interaction of Cr^3+^ with ATNA/Nafion/GCE: (**a**) the *I-V* response without Cr^3+^; (**b**) the *I-V* response with Cr^3+^; (**c**) the comparison of the I-V response with and without Cr^3+^; and (**d**) the probable π–π* interaction between the Cr^3+^ ions and ATNA.

**Figure 7:**
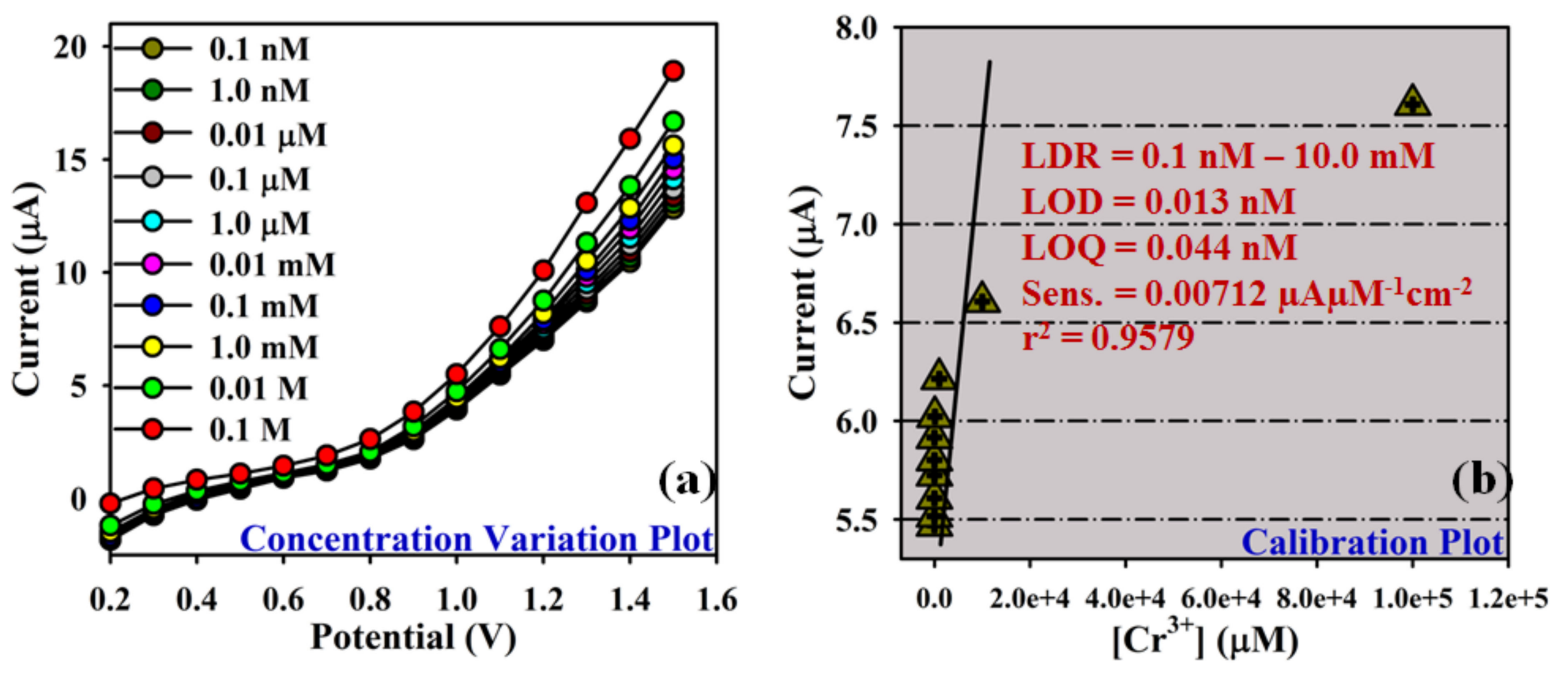
Optimization of the newly designed Cr^3+^ sensor: (**a**) the concentration variation plot of Cr^3+^ (0.1 M to 0.1 nM); and (**b**) the calibration plot (at + 1.1 V) of ATNA/Nafion/GCE.

The ATNA Schiff base accommodates highly electronegative *e*^−^-donating atoms, which were also confirmed by structure characterization through single-crystal X-ray diffraction analysis (Figure 1 and Figure 2). It donates the electron pairs to Cr^3+^ ions, which results in a change in (*I-V*) response as presented in the proposed mechanism (Scheme 4). It was also shown from the interference study that the ATNA Schiff base has a high affinity with Cr^3+^ ions in the company of other heavy metal cations. Moreover, the (I-V) response of newly designed ATNA/Nafion/GCE (as a selective as well as sensitive Cr^3+^ cationic electrochemical sensor) against the potential applied (0 −1.5 V) become functional in the presence of Cr^3+^ ions. The current responses in the presence and absence of Cr^3+^ ions are shown in Scheme 4a–c. Upon adding a small amount (25.0 µL of 0.1 µM) of the target analyte (Cr^3+^) in a PBS medium of pH = 7 through a digital micropipette, a marginal increase in the current response was observed due to the small surface of the modified GCE that was occupied by the Cr^3+^ ions. Therefore, the surface reaction continues and does not stop here. It proceeds continuously by increasing the concentration of the Cr^3+^ analytes onto the flat surface of the modified GCE in the system (π–π* interaction), which gives the regular increment of a current response as a function of our target analytes. We can get a better idea from the concentration plot, Figure 7a, which gives us an idea about the increase in the current response by increasing the concentration of Cr^3+^ in the system. Thus, the observed increase in the current response was in accordance with the occupation of the coated surface of modified GCE with the target analytes (Cr^3+^) and at the end, the coated surface became saturated with Cr^3+^ ions as shown in Scheme 4d. Furthermore, it caused an increase in the π–π* interaction between the functional groups of ATNA and Cr^3+^ ions, which is responsible for the change in the current response, Scheme 4b. The same phenomena based on the above stipulated proposed mechanism for the detection of toxic chemicals have also been cited in the literature [59,60].

In order to optimize the newly designed ATNA/Nafion/GCE as sensitive as well as a selective and efficient Cr^3+^ cationic electro-chemical sensor, a concentration variation plot was drawn at the different concentration range of Cr^3+^ analytes (0.1 M to 0.1 nM) in addition to the calibration curve at +1.1 V from for above discussed concentration range of Cr^3+^ analytes, Figure 7a,b. It was observed that the current responses of ATNA/Nafion/GCE, as a selective cationic electrochemical Cr^3+^ sensor, progressively increased against the potential applied, from a lower to a higher value, as a function of Cr^3+^ concentration at standard temperature and pressure (STP), Figure 7a. Moreover, analytical parameters such as the LDR, LOD, LOQ, sensitivity, and the regression coefficient (r^2^) were calculated from the calibration plot at +1.1 V and these were found to be as (0.1 nM–10 mM), 0.013 nM, 0.044 nM, 0.0071202 µAµM^−1^cm^−2^ and 0.9579, respectively, Figure 7b. 

**Figure 8:**
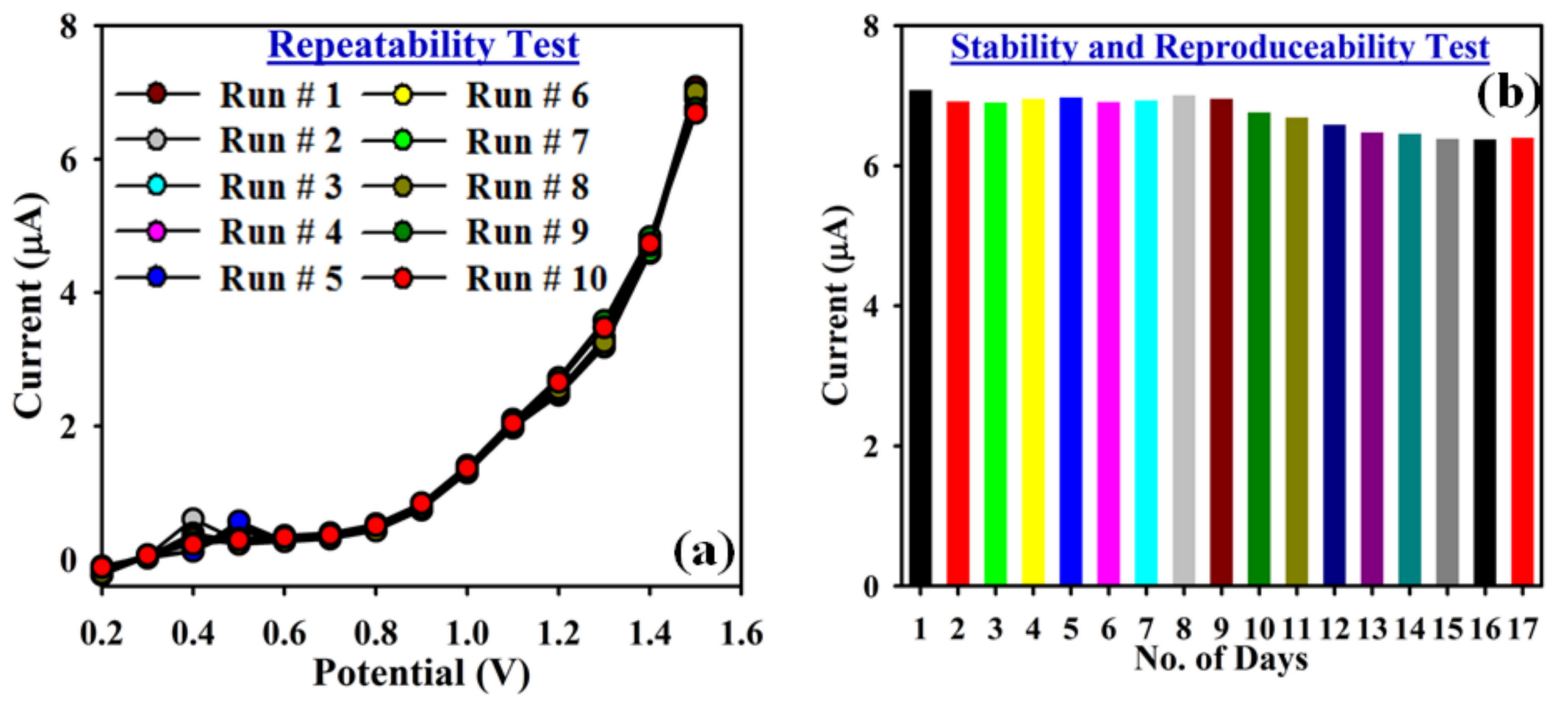
Repeatability and stability study: (**a**) the repeatability rest; and (**b**) the stability test of newly modified GCE (ATNA/Nafion/GCE as an efficient and sensitive Cr^3+^ cationic electrochemical sensor).

Repeatability and response time, in addition to stability, were also checked in order to validate the newly designed ATNA/Nafion/GCE as an efficient electrochemical sensor. The repeatability test was conducted with successive nine to ten measurements of our target analyte (Cr^3+^), which were taken as 0.25 µL of 0.1 µM of Cr^3+^ cations in each measurement, Figure 8a. In addition, a stability (reproducibility) test was also conducted and examined up to 17 days. It was found to be very stable with no significant change in the I-V response in addition to electrode poisoning and erosion up to 9 days, Figure 8b. 

**Figure 9:**
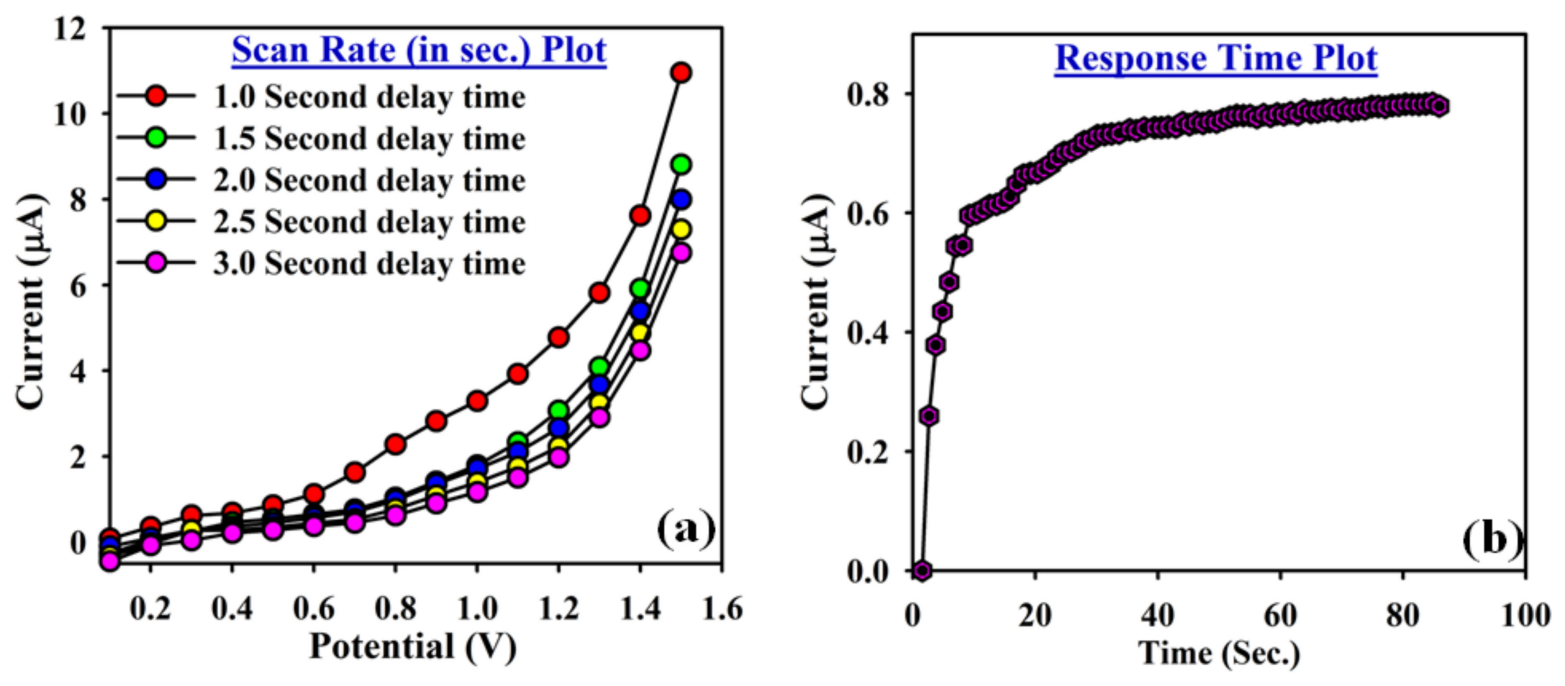
Response time: (**a**) the evaluation of the scan rates against the potential applied (0–1.5 V); and (**b**) the response time from 10 to 20 s to reach the saturated steady state.

Similarly, the response time of the modified GCE was recorded (as current (µA) versus (s) time), and it was found to be very sensitive with a fast response in a short time that was from 10 to 20 s. It was also noticed that it gave a good current response at pH = 7 against the potential applied (0–1.5 V) at a 1.0 s delay time as the scan rate, compared to the other scan rates in the presence of Cr^3+^, Figure 9a,b.

The above conducted experiments enlighten us that the sensitivity of the newly designed ATNA/Nafion/GCE as a selective Cr^3+^ cationic electrochemical sensor is correlated with the adsorption as well as the absorption ability of the target analyte (Cr^3+^) on to the surface of the modified GCE. It also depends on the affinity of the homobifunctional tridentate Schiff base with Cr^3+^ ions in addition to the high electronic communication feature which exists among the active sites of ATNA and Cr^3+^ ions. Moreover, the sensitivity and detection limits were also in good accordance as compared to the previous methods that have been reported in the literature, Table 4. This current study also introduces a very simple, reliable approach to a significant extent for the optimistic detection of toxic chemicals in an aqueous media/solution in an ideal environmental and different care fields by using the newly designed ATNA as a tridentate homobifunctional disulphide Schiff base as a cationic electrochemical sensor. The obtained sensor analytical results exhibited the higher sensitivity and lower detection limit as well as the short response time as compared to other sensors fabricated with other materials for heavy metal ions in recent reports [61,62,63,64]. This is an initial report for the qualitative as well as the quantitative detection of Cr^3 +^ ions by the electrochemical current-potential (I-V) approach based on newly modified GCE (ATNA/Nafion/GCE).

### 3.4. Real Sample Analysis

The analysis of the real samples was also conducted by using this new proposed I-V approach through a standard addition method [15] so as to validate the newly modified GCE as an efficient and selective cationic electrochemical sensor for Cr^3+^ ion. A fixed quantity i.e., 25.0 µL of each sample was investigated in PBS (0.2 M, pH = 7.0). Table 5 shows the results, which indicate that this proposed technique with newly modified GCE is also reliable for the investigation of Cr^3+^ in real samples.

## 4. Conclusions

A new electrochemical sensor for the selective detection of Cr^3+^ in aqueous solution based on homobifunctional tridentate disulfide Schiff base was presented for the first time. The conformation of ATNA as confirmed by the single crystal X-ray diffractions indicate that the imine bond (C=N) in both parts in the molecule is trans, favoring the molecule to stay in the most stable form. The molecule was stabilized by the O–H…N type intramolecular hydrogen bonding interactions. The ATNA/Nafion/GCE as an efficient and sensitive electrochemical sensor for the detection of toxic cations in an aqueous solution by means of the electrochemical (I-V) approach was investigated. It was found to be very stable as well as sensitive against the Cr^3+^ ions in a short response time with a detection limit as low as 0.013 nM. Therefore, this study offers the new idea that can be used for the invention of newly non-reported cationic electrochemical sensors based on multidentate ligands or chelating agents for the investigation of heavy metals cations in commercially.

## Figures and Tables

**Table 1:** Data and structure refinement for ATNA.

CCDC Code (Cambridge Crystallographic Data Centre)	1953254
Empirical formula	C_34_H_24_N_2_O_2_S_2_
Formula weight	556.67
Temperature/K	296(2)
Crystal system	triclinic
Space group	P-1
a/Å	8.6843(5)
b/Å	11.8282(6)
c/Å	13.5159(9)
α/°	78.828(5)
β/°	82.291(5)
γ/°	86.223(4)
Volume/Å^3^	1348.59(14)
Z	2
ρ_calc_g/cm^3^	1.371
μ/mm^−1^	0.233
F(000)	580.0
Crystal size/mm^3^	0.22 × 0.17 × 0.12
Radiation	MoKα (λ = 0.71073)
2θ range for data collection/°	5.942 to 58.364
Index ranges	−11 ≤ h ≤ 7, −15 ≤ k ≤ 15, −17 ≤ l ≤ 18
Reflections collected	12,053
Independent reflections	6334 (R_int_ = 0.0338, R_sigma_ = 0.0553)
Data/restraints/parameters	6334/0/361
Goodness-of-fit on F^2^	1.031
Final R indexes indexes (I>=2σ (I))	R_1_ = 0.0538, wR_2_ = 0.1211
Final R indexes (all data)	R_1_ = 0.1039, wR_2_ = 0.1544
Largest diff. peak/hole / e Å^−3^	0.23/−0.27

**Table 2:** Hydrogen bonds for ATNA.

D	H	A	d(D-H)/Å	d(H-A)/Å	d(D-A)/Å	D-H-A/°
O1	H1O	N1	0.82	1.85	2.575(3)	146.8
O2	H2O	N2	0.82	1.85	2.574(3)	147.4
O2	H2O	S2	0.82	2.74	3.410(2)	139.7

**Table 3:** Interference effect of the various cations with ATNA/Nafion/GCE.

Metal Ions	Observed Current (µA)				Interference Effect (%)	^*^SD (n= 3)	^#^RSD (%) (n = 3)
	R1	R2	R3	Average			
**Cr^3+^**	14.9618	14.8241	15.1423	14.9760	100	0.1595	1.07
**Al^3+^**	5.2456	6.1323	5.8625	5.7468	38	0.4545	7.91
**Ce^2+^**	7.1919	7.2031	7.1879	7.1943	48	0.0078	0.11
**Co^2+^**	6.1792	6.2955	6.0095	6.1614	41	0.1438	2.33
**Cu^2+^**	6.5441	6.6192	6.8994	6.6875	44	0.1872	2.80
**Ga^3+^**	6.3739	6.2084	6.3215	6.3012	42	0.0845	1.34
**Hg^2+^**	6.5512	6.4989	6.3003	6.4501	43	0.1323	2.05
**Mn^2+^**	4.0958	4.3853	4.8554	4.4455	29	0.3833	8.62
**Pb^2+^**	5.9285	5.7969	5.6714	5.7989	38	0.1285	2.22
**Y^3+^**	5.8275	6.2791	6.3461	6.1509	41	0.2820	4.59

Interference effect of Cr^3+^ is considered to be 100%; R = reading; ^*^SD = standard deviation; and ^#^RSD = relative standard deviation.

**Table 4:** Comparison of the proposed *I-V* method with different previously reported analytical methods for the detection of Cr^3+^.

Methods	Material	Sensitivity	^*^LDR	^#^LOD	^@^LOQ	Ref.
**Amperometric**	Tyrosinase biosensor	–	2.0 × 10^−4^ M	500.0 nM	–	[48]
**Capillary electrophoresis**	1,2-Cyclohexanediaminetetraacetic acid (CDTA)	–	0.0 M–0.0019 M	961.6 nM	–	[40]
**Capillary electrophoresis**	Hexamolybdochromate	–	5 × 10^−6^–1 × 10^−5^ M	2000 nM	–	[41]
**Chemiluminescence**	Ethylene diamine tetra acetate (EDTA)	–	0.0–1 × 10^−6^ M	0.5 nM	–	[42]
**Cyclic voltammetry&amperometry**	Gold nanoparticle-decorated titania nanotube arrays	6.91 µAµM^−1^	0.10 µM–105 µM	0.03 µM	–	[45]
**Electrothermal atomic absorption spectrometric**	Nano TiO_2_	–	1 × 10^−3^–0.5 M	0.11 nM	–	[27]
**Thin-layer X-ray fluorescence spectrometry**	Solid-phase hydrous ferric hydroxide (HFO)	–	0.0–1.0 µM	16.9 nM	–	[36]
**High-performance liquid chromatography with diode-array detection**	Ammonium pyrrolidinedithiocarbamate (APDC)	–	190 nM–0.76 mM	76,900–134,000 nM	–	[28]
**Inductively Coupled Plasma** **Atomic-emission Spectrometry**	Micro-column of activated alumina	–	0.0–1.9 × 10^−5^ M	26.0 nM	–	[26]
**Electrochemical I-V method**	**ATNA/Nafion/GCE**	**0.0071202 µAµM^−1^cm^−2^**	**1.0 nM–0.01 M**	**0.013 nM**	**0.04 nM**	**This work**

*LDR = linear dynamic ranger; ^#^LOD = limit of detection; ^@^LOQ = limit of quantification

**Table 5:** Real sample analysis of Cr^3+^ in various environmental samples.

Real Samples	Amount of 3-CP Added	No. of Readings	Measured Response in (µA)	% Recovery	Mean (% Recovery)	SD	RSD	SEM
**Cr^3+^**	0.1 µM, 25 µL	-	13.6808	100	-	-	-	-
**Plastic baby feeding bottle**	0.1 µM, 25 µL	R1	13.2376	96.7	**91.2**	4.765	5.22	2.75
		R2	12.2129	89.3				
		R3	12.0183	87.8				
**Industrial effluent**	0.1 µM, 25.0 µL	R1	14.9859	109.5	**106.7**	3.143	2.95	1.81
		R2	14.6821	107.3				
		R3	14.1258	103.3				
**Sea water**	0.1 µM, 25.0 µL	R1	12.1036	88.5	**88.1**	1.833	2.08	1.05
		R2	11.7748	86.1				
		R3	12.2757	89.7				
**Tap water**	0.1 µM, 25.0 µL	R1	13.2457	96.8	**97.9**	1.850	1.89	1.06
		R2	13.6980	100.1				
		R3	13.2682	97.0				

SD = standard deviation; RSD = relative standard deviation; SEM = standard error of mean

## Supplementary Materials

The following are available online at https://www.mdpi.com/1996-1944/13/12/2695/s1.
