# Supplementary material for: A New Cr3+ Electrochemical Sensor Based on ATNA/Nafion/Glassy Carbon Electrode
Source: Materials (Basel). 2020 Jun 12;13(12):2695. doi: 10.3390/ma13122695 (PMC7345133; doi:10.3390/ma13122695)
Supplement: Supplementary file 1 [file materials-13-02695-s001.pdf]

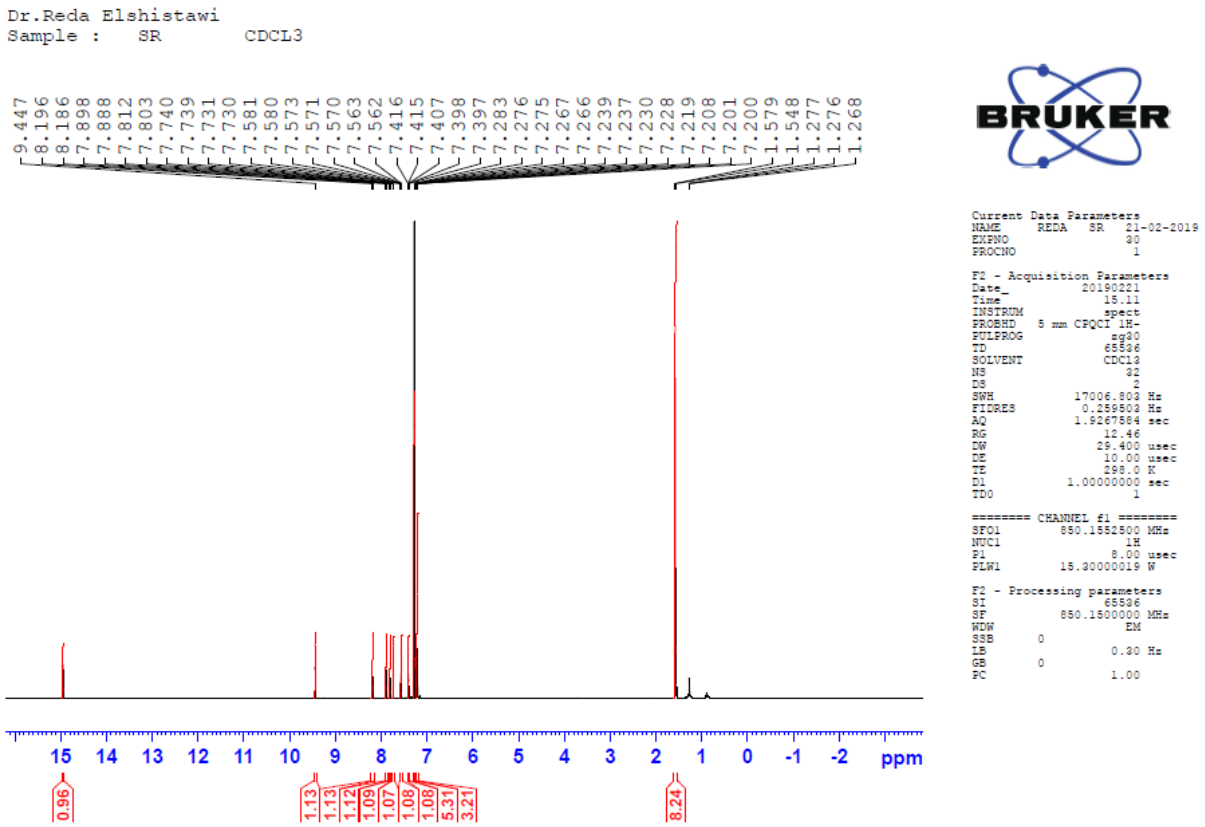
Dr.Reda Elshistawi
Sample : SR CDCL3
9.447 8.196 8.186 7.898 7.888 7.812 7.803 7.740 7.739 7.731 7.730 7.581 7.580 7.573 7.571 7.570 7.563 7.562 7.416 7.415 7.407 7.398 7.397 7.283 7.276 7.275 7.267 7.266 7.239 7.237 7.230 7.228 7.219 7.208 7.201 7.200 1.579 1.548 1.277 1.276 1.268
BRUKER
Current Data Parameters
NAME REDA SR 21-02-2019
EXPNO 30
PROCNO 1
F2 - Acquisition Parameters
Date_ 20190221
Time 15.11
INSTRUM spect
PROBHD 5 mm CPQCI 1H-
PULPROG zg30
TD 65536
SOLVENT CDCl3
NS 32
DS 2
SWH 17006.803 Hz
FIDRES 0.259503 Hz
AQ 1.9267584 sec
RG 12.46
DW 29.400 usec
DE 10.00 usec
TE 298.0 K
D1 1.00000000 sec
TD0 1
======== CHANNEL f1 ========
SFO1 850.1552500 MHz
NUC1 1H
P1 8.00 usec
PLW1 15.30000019 W
F2 - Processing parameters
SI 65536
SF 850.1500000 MHz
WDW EM
SSB 0
LB 0.30 Hz
GB 0
PC 1.00
15 14 13 12 11 10 9 8 7 6 5 4 3 2 1 0 -1 -2 ppm
0.96 1.13 1.13 1.12 1.09 1.07 1.08 1.08 5.31 3.21 8.24

S1.

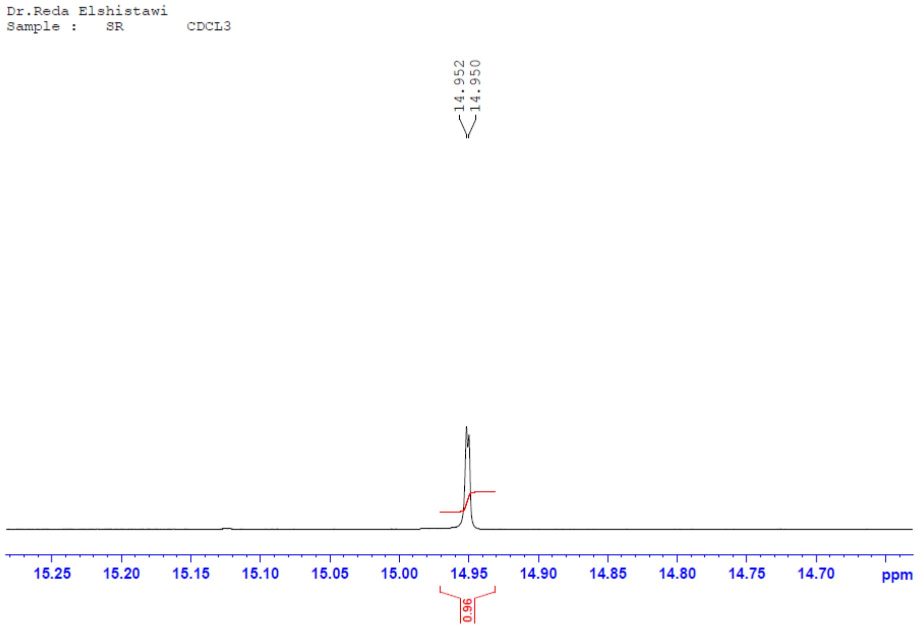
Dr.Reda Elshistawi
Sample : SR CDCL3
14.952
14.950
15.25
15.20
15.15
15.10
15.05
15.00
14.95
14.90
14.85
14.80
14.75
14.70
ppm
0.96

S2.

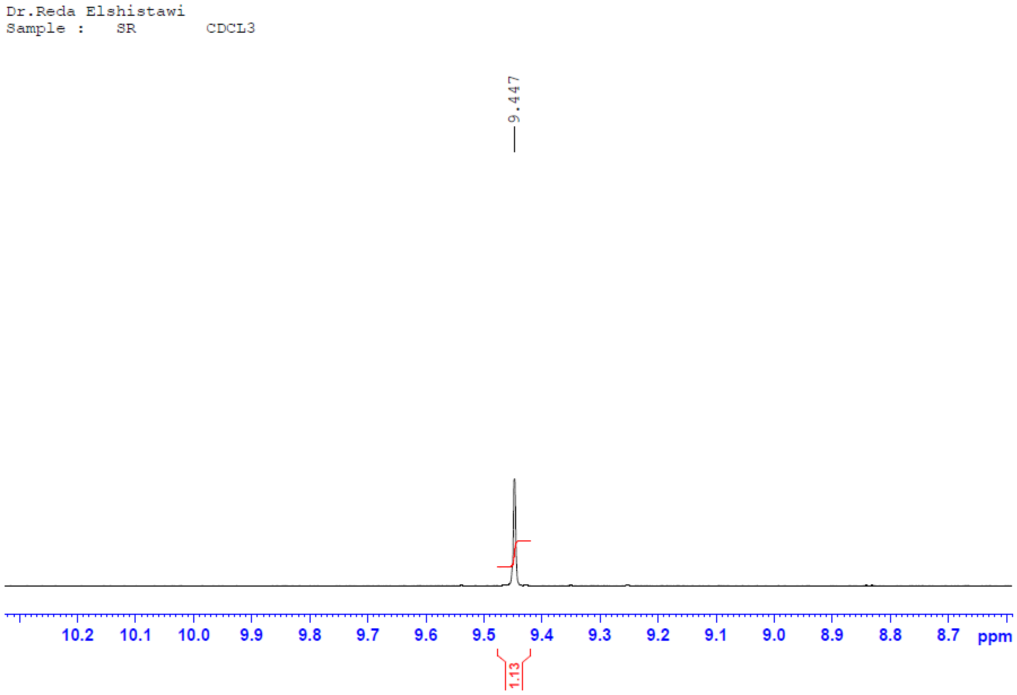
Dr.Reda Elshistawi
Sample : SR CDCL3
9.447
10.2
10.1
10.0
9.9
9.8
9.7
9.6
9.5
9.4
9.3
9.2
9.1
9.0
8.9
8.8
8.7
ppm
1.13

S3.

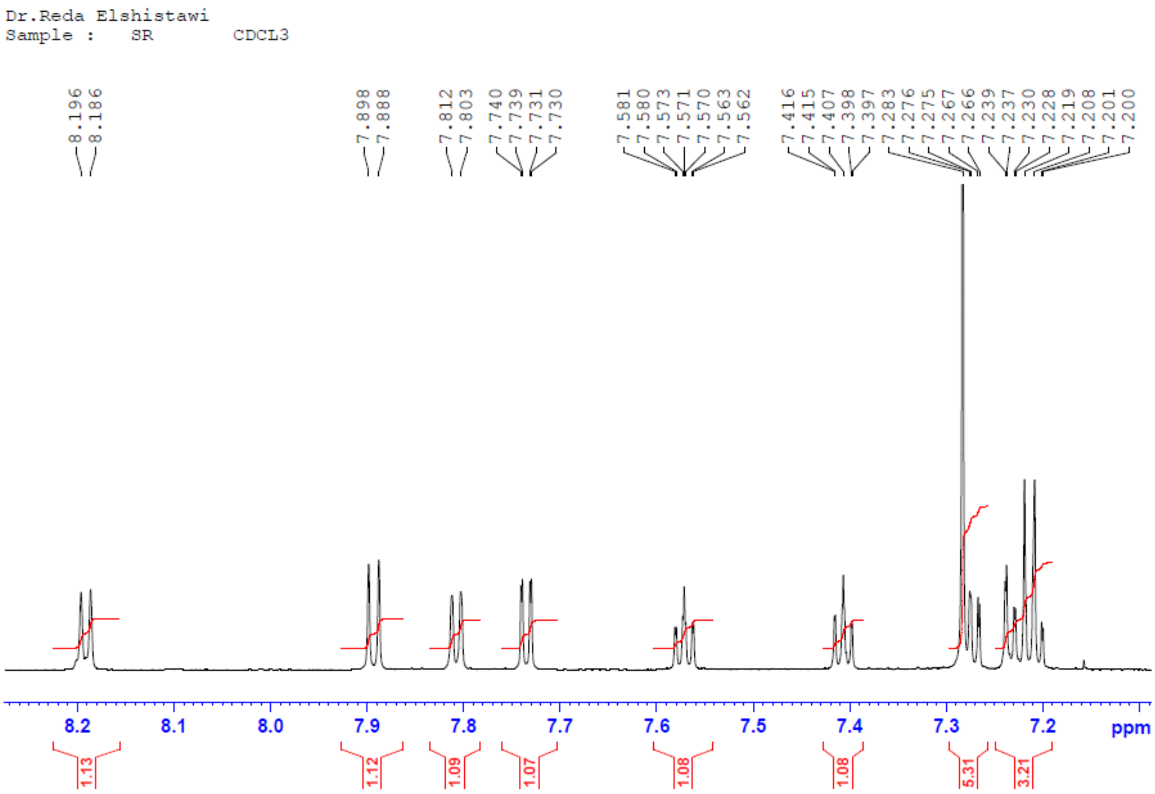

S4.

Figure S1. Proton NMR of ATNA (S1–S4).

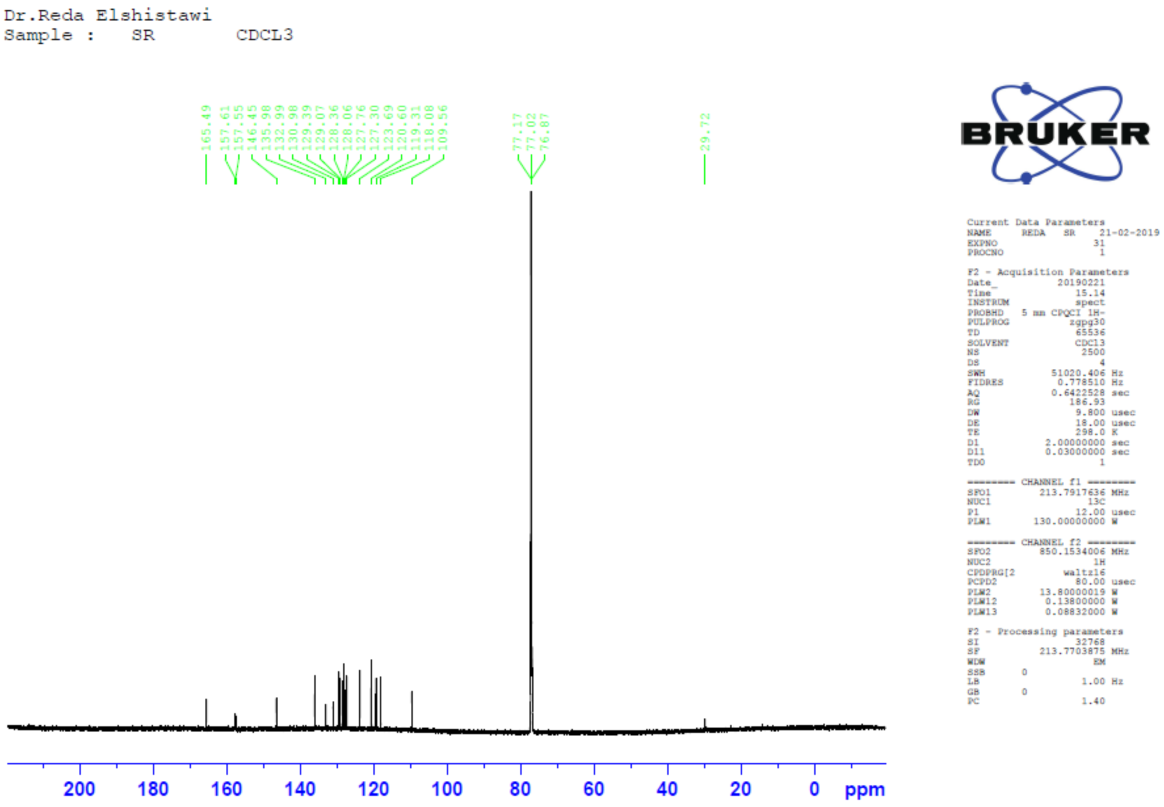

Dr.Reda Elshistawi
Sample : SR CDCL3
165.49
157.61
157.55
146.45
135.98
132.99
130.98
129.39
129.07
128.36
128.06
127.76
127.30
123.69
120.60
119.31
118.08
109.56
77.17
77.02
76.87
29.72
Current Data Parameters
NAME REDA SR 21-02-2019
EXPNO 31
PROCNO 1
F2 - Acquisition Parameters
Date_ 20190221
Time 15.14
INSTRUM spect
PROBHD 5 mm CPQCI 1H-
PULPROG zgpg30
TD 65536
SOLVENT CDCl3
NS 2500
DS 4
SWH 51020.406 Hz
FIDRES 0.778510 Hz
AQ 0.6422528 sec
RG 186.93
DW 9.800 usec
DE 18.00 usec
TE 298.0 K
D1 2.00000000 sec
D11 0.03000000 sec
TD0 1
======== CHANNEL f1 ========
SFO1 213.7917636 MHz
NUC1 13C
P1 12.00 usec
PLW1 130.00000000 W
======== CHANNEL f2 ========
SFO2 850.1534006 MHz
NUC2 1H
CPDPRG[2 waltz16
PCPD2 80.00 usec
PLW2 13.80000019 W
PLW12 0.13800000 W
PLW13 0.08832000 W
F2 - Processing parameters
SI 32768
SF 213.7703875 MHz
WDW EM
SSB 0
LB 1.00 Hz
GB 0
PC 1.40
200 180 160 140 120 100 80 60 40 20 0 ppm

S5.

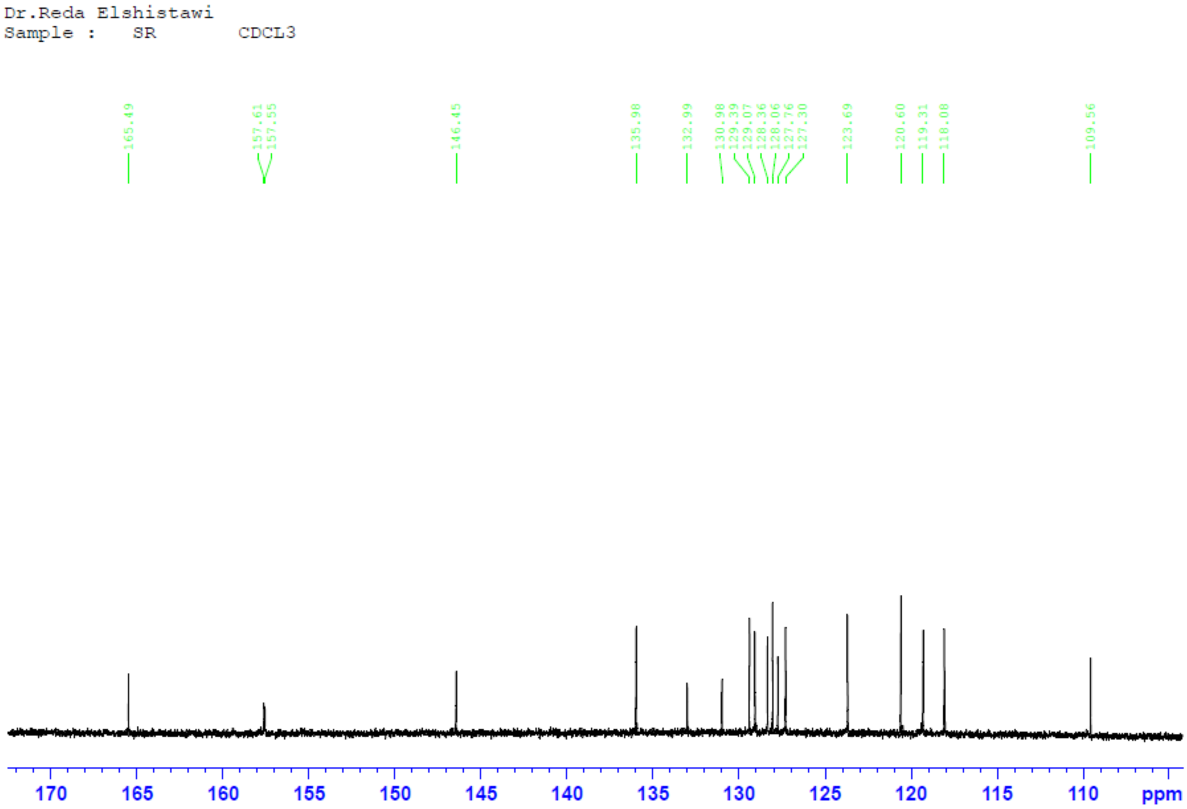

S6.

Figure S2. Carbon NMR of ATNA (S5 and S6).

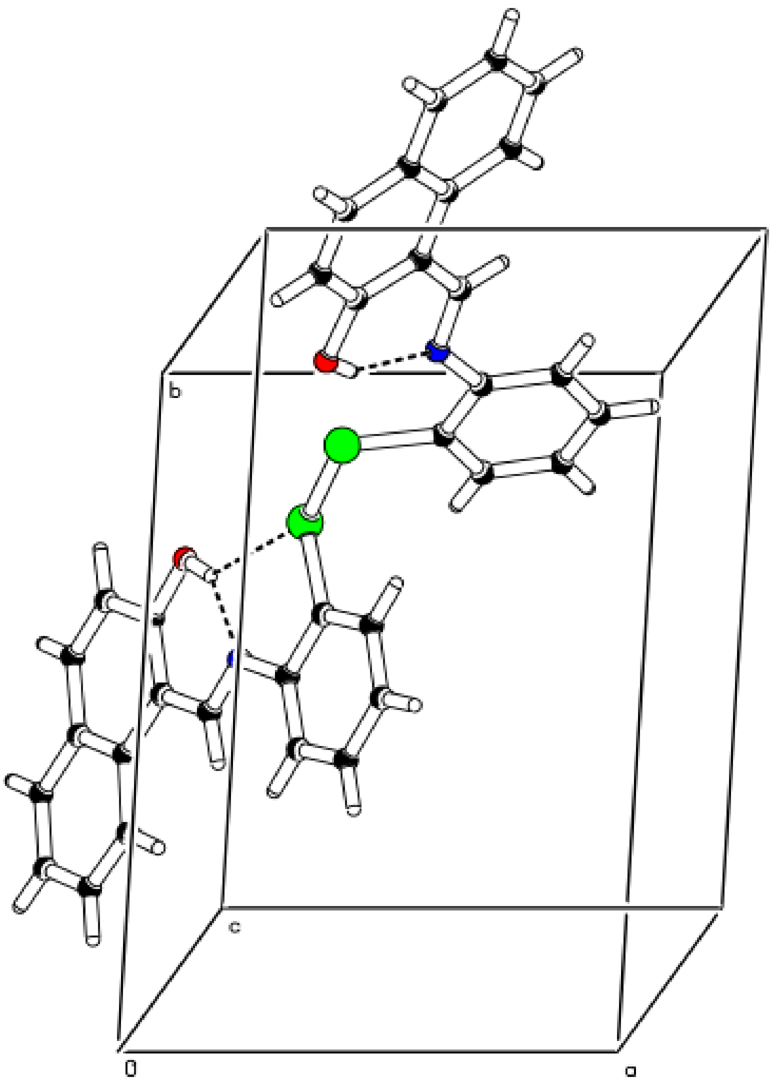

Figure S3. A unit cell diagram of ATNA showing Intramolecular hydrogen bonding using dashed lines.

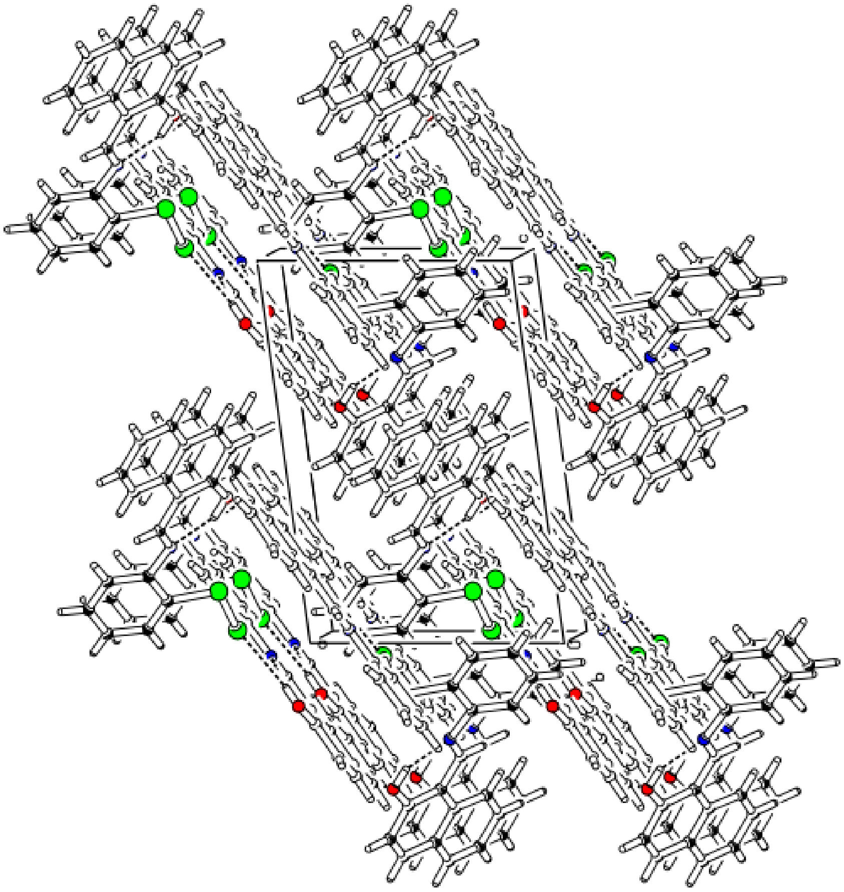

Figure S4. A packing diagram where planes are parallel to each other and showing the possibility of π-π interactions.

Table S1. Bond Lengths for ATNA.

Atom	Atom	Length/Å	Atom	Atom	Length/Å
C1	C2	1.400(3)	C18	C19	1.392(3)
C1	C11	1.441(3)	C18	S2	1.787(2)
C1	C6	1.450(3)	C19	C20	1.384(3)
C2	O1	1.342(3)	C19	N2	1.416(3)
C2	C3	1.397(4)	C20	C21	1.378(4)
C3	C4	1.351(4)	C21	C22	1.373(4)
C4	C5	1.409(4)	C22	C23	1.365(3)
C5	C6	1.409(4)	C24	N2	1.282(3)
C5	C10	1.425(4)	C24	C25	1.433(3)
C6	C7	1.402(3)	C25	C26	1.398(3)
C7	C8	1.366(4)	C25	C30	1.446(3)
C8	C9	1.393(4)	C26	O2	1.344(3)
C9	C10	1.346(4)	C26	C27	1.411(4)
C11	N1	1.286(3)	C27	C28	1.341(4)
C12	C13	1.397(3)	C28	C29	1.414(4)
C12	C17	1.402(3)	C29	C34	1.409(4)
C12	N1	1.407(3)	C29	C30	1.419(3)
C13	C14	1.385(3)	C30	C31	1.405(3)
C13	S1	1.780(3)	C31	C32	1.377(3)
C14	C15	1.386(4)	C32	C33	1.380(4)
C15	C16	1.377(4)	C33	C34	1.355(4)
C16	C17	1.362(4)	S1	S2	2.0234(10)
C18	C23	1.384(3)			

Table S2. Bond Angles for ATNA.

Atom	Atom	Atom	Angle/°	Atom	Atom	Atom	Angle/°
C2	C1	C11	120.0(2)	C19	C18	S2	115.80(18)
C2	C1	C6	118.5(2)	C20	C19	C18	118.5(2)
C11	C1	C6	121.4(2)	C20	C19	N2	125.1(2)
O1	C2	C3	116.5(2)	C18	C19	N2	116.4(2)
O1	C2	C1	122.5(2)	C21	C20	C19	120.8(2)
C3	C2	C1	121.0(3)	C22	C21	C20	119.7(2)
C4	C3	C2	120.3(3)	C23	C22	C21	120.8(3)
C3	C4	C5	121.9(3)	C22	C23	C18	119.7(2)
C6	C5	C4	119.3(3)	N2	C24	C25	122.8(2)
C6	C5	C10	119.5(3)	C26	C25	C24	120.2(2)
C4	C5	C10	121.3(3)	C26	C25	C30	118.4(2)
C7	C6	C5	117.6(2)	C24	C25	C30	121.4(2)
C7	C6	C1	123.4(2)	O2	C26	C25	122.0(2)
C5	C6	C1	119.0(2)	O2	C26	C27	117.2(2)
C8	C7	C6	121.9(3)	C25	C26	C27	120.8(2)
C7	C8	C9	120.1(3)	C28	C27	C26	120.5(3)
C10	C9	C8	120.3(3)	C27	C28	C29	122.2(3)
C9	C10	C5	120.7(3)	C34	C29	C28	122.0(3)
N1	C11	C1	122.3(2)	C34	C29	C30	119.3(3)
C13	C12	C17	118.5(2)	C28	C29	C30	118.6(2)
C13	C12	N1	116.8(2)	C31	C30	C29	117.2(2)
C17	C12	N1	124.6(2)	C31	C30	C25	123.3(2)
C14	C13	C12	120.1(3)	C29	C30	C25	119.5(2)
C14	C13	S1	124.5(2)	C32	C31	C30	121.3(3)
C12	C13	S1	115.39(19)	C31	C32	C33	121.2(3)
C13	C14	C15	120.2(3)	C34	C33	C32	119.1(3)
C16	C15	C14	119.6(3)	C33	C34	C29	121.9(3)
C17	C16	C15	120.9(3)	C11	N1	C12	122.0(2)
C16	C17	C12	120.6(3)	C24	N2	C19	122.80(19)
C23	C18	C19	120.4(2)	C13	S1	S2	105.28(9)
C23	C18	S2	123.76(19)	C18	S2	S1	104.91(9)
